# Iron or sulfur respiration—an adaptive choice determining the fitness of a natronophilic bacterium *Dethiobacter alkaliphilus* in geochemically contrasting environments

**DOI:** 10.3389/fmicb.2023.1108245

**Published:** 2023-07-14

**Authors:** Daria G. Zavarzina, Alexander Yu Merkel, Alexandra A. Klyukina, Ivan M. Elizarov, Valeria A. Pikhtereva, Vyacheslav S. Rusakov, Nataliya I. Chistyakova, Rustam H. Ziganshin, Alexey A. Maslov, Sergey N. Gavrilov

**Affiliations:** ^1^Winogradsky Institute of Microbiology, FRC Biotechnology, Russian Academy of Sciences, Moscow, Russia; ^2^Faculty of Biology, Lomonosov Moscow State University, Moscow, Russia; ^3^Faculty of Physics, Lomonosov Moscow State University, Moscow, Russia; ^4^Shemyakin-Ovchinnikov Institute of Bioorganic Chemistry, Russian Academy of Sciences, Moscow, Russia; ^5^Faculty of Geology, Lomonosov Moscow State University, Moscow, Russia

**Keywords:** *Dethiobacter alkaliphilus*, soda lakes, serpentinites, multiheme cytochromes, sulfur/thiosulfate reduction, microbial iron cycling

## Abstract

Haloalkaliphilic microorganisms are double extremophiles functioning optimally at high salinity and pH. Their typical habitats are soda lakes, geologically ancient yet widespread ecosystems supposed to harbor relict microbial communities. We compared metabolic features and their determinants in two strains of the natronophilic species *Dethiobacter alkaliphilus*, the only cultured representative of the class “*Dethiobacteria*” (*Bacillota*). The strains of *D. alkaliphilus* were previously isolated from geographically remote Mongolian and Kenyan soda lakes. The type strain AHT1^T^ was described as a facultative chemolithoautotrophic sulfidogen reducing or disproportionating sulfur or thiosulfate, while strain Z-1002 was isolated as a chemolithoautotrophic iron reducer. Here, we uncovered the iron reducing ability of strain AHT1^T^ and the ability of strain Z-1002 for thiosulfate reduction and anaerobic Fe(II) oxidation. Key catabolic processes sustaining the growth of both *D. alkaliphilus* strains appeared to fit the geochemical settings of two contrasting natural alkaline environments, sulfur-enriched soda lakes and iron-enriched serpentinites. This hypothesis was supported by a meta-analysis of *Dethiobacterial* genomes and by the enrichment of a novel phylotype from a subsurface alkaline aquifer under Fe(III)-reducing conditions. Genome analysis revealed multiheme *c*-type cytochromes to be the most probable determinants of iron and sulfur redox transformations in *D. alkaliphilus*. Phylogeny reconstruction showed that all the respiratory processes in this organism are likely provided by evolutionarily related early forms of unconventional octaheme tetrathionate and sulfite reductases and their structural analogs, OmhA/OcwA Fe(III)-reductases. Several phylogenetically related determinants of anaerobic Fe(II) oxidation were identified in the Z-1002 genome, and the oxidation process was experimentally demonstrated. Proteomic profiling revealed two distinct sets of multiheme cytochromes upregulated in iron(III)- or thiosulfate-respiring cells and the cytochromes peculiar for Fe(II) oxidizing cells. We suggest that maintaining high variation in multiheme cytochromes is an effective adaptive strategy to occupy geochemically contrasting alkaline environments. We propose that sulfur-enriched soda lakes could be secondary habitats for *D. alkaliphilus* compared to Fe-rich serpentinites, and that the ongoing evolution of *Dethiobacterales* could retrace the evolutionary path that may have occurred in prokaryotes at a turning point in the biosphere’s history, when the intensification of the sulfur cycle outweighed the global significance of the iron cycle.

## Introduction

Modern soda lakes, defined by high salinity and pH, occur worldwide and are thought to harbor relict microbial communities (Zavarzin, 1993; Zhilina and Zavarzin, 1994). Despite several extreme parameters, soda lakes are characterized by high productivity and contain fully functional and diverse haloalkaliphilic microbial communities that drive the biogeochemical cycles of carbon, nitrogen, and sulfur (Jones et al., 1998; Zavarzin et al., 1999; Sorokin et al., 2011, 2014a). In modern soda lakes, the sulfur cycle is one of the most active biogeochemical processes. Redox transformations of inorganic sulfur compounds are energetically efficient enough for microorganisms to cope with costly life under polyextreme conditions. An important phenomenon observed in the sediments of Kulunda soda lakes (Altai region, Russia) is that sulfidogens using elemental sulfur and thiosulfate as electron acceptors are more active there than sulfate reducers despite the higher abundance of sulfates in the ecosystem (Sorokin et al., 2011). Inspite of the predominance of the sulfur cycle, several species and genera of iron reducers have been isolated from soda lakes and their wide distribution in these ecosystems has been further reported (Zavarzina et al., 2016). Importantly, most dissimilatory alkaliphilic iron reducers are capable of using sulfur as an alternative electron acceptor, just as many alkaliphilic sulfidogens have been shown to reduce Fe(III) (Zavarzina et al., 2018).

*Dethiobacter alkaliphilus*, the only cultured representative of the genus *Dethiobacter*, was isolated from mixed anaerobic sediments of northeastern Mongolian soda lakes, and was described as a sulfur- and thiosulfate-reducing, facultatively chemolithoautotrophic sulfidogen utilizing H_2_ and a range of organic electron donors, strain AHT1^T^ (Sorokin et al., 2008). The organism is also capable of sulfur or thiosulfate disproportionation (Poser et al., 2013, 2016). The 16S rRNA gene-based phylogenetic reconstruction placed the first isolate of *D. alkaliphilus* within a deep lineage of *Firmicutes* (now *Bacillota*) related to the order S*yntrophomonadales* and syntrophic acetate-oxidizing haloalkaliphiles from soda lakes, with *Ca.* Contubernalis alkalaceticus (Zhilina et al., 2005) and *Ca.* Synthrophonatronum acetioxidans (Sorokin et al., 2014b) as the closest relatives. Further phylogenomic reconstruction based on 120 single-copy conservative markers (Parks et al., 2018) rooted *D. alkaliphilus* as a separate class “*Dethiobacteria*,” order “*Dethiobacterales*,” and family “*Dethiobacteraceae*” (Sorokin and Merkel, 2019). Strain Z-1002 belonging to the same species, was isolated from the sediments of a hypersaline soda lake Magadi in Kenya (Zavarzina et al., 2018) on the selective medium containing synthesized ferrihydrite (SF) as the sole electron acceptor and formate or molecular hydrogen as the energy source. Thus, the two cultured strains AHT1^T^ and Z-1002 of the single species of the class “*Dethiobacteria*” were isolated as sulfur/thiosulfate- and iron-reducers, respectively, from the sediments of soda lakes located on two different continents.

Interestingly, *Dethiobacter*-related phylotypes have been previously detected exclusively in alkaline environments (pH ≥ 7.5) of two different types, the sediments of soda or meromictic lakes, and subsurface ecosystems affected by serpentinization processes. Representatives of *Dethiobacter* genus were detected in the sediments of a meromictic Mahoney lake, in soda lakes Mono, Van and those of the Kulunda Steppe (Hamilton et al., 2016; Edwardson and Hollibaugh, 2017, 2018; Vavourakis et al., 2018; Ersoy Omeroglu et al., 2021), as well as in several serpentinizing ophiolites, serpentinization-based subseafloor ecosystems, and terrestrial subsurface environments (Brazelton et al., 2013; Suko et al., 2013; Suzuki et al., 2013; Tiago and Veríssimo, 2013; Crespo-Medina et al., 2014; Glaring et al., 2015; López-López et al., 2015; Postec et al., 2015; Woycheese et al., 2015; Purkamo et al., 2016, 2017; Pisapia et al., 2017; Sabuda et al., 2020, 2021). Furthermore, *Dethiobacter* was highly enriched in microcosms with serpentinizing groundwater in the absence of any external nutrients (Crespo-Medina et al., 2014; Purkamo et al., 2017). Serpentinization is a widespread geochemical phenomenon involving the aqueous alteration of ultramafic rocks, containing the Fe-rich mineral olivine [(Mg,Fe)_2_SiO_4_], to serpentine [(Mg,Fe,)_2-3_(Si,Fe)_2_O_5_(OH)_4_]. The process results in the formation of a highly alkaline (pH > 10) and strongly reducing, H_2_-rich fluid, which creates thermodynamically favorable environmental conditions for abiotic organic synthesis from slab-derived inorganic carbon (McCollom and Bach, 2009). Serpentinization occurs in numerous settings on Earth, including subduction zones, mid-ocean ridges, and ophiolites and is rooted far back in the Earth’s geologic history, potentially contributing to the origin and early evolution of life (Russell et al., 2010; Schrenk et al., 2013). In this view, the ecological fitness of *Dethiobacter* correlates with the putative conditions of some early Earth’s ecosystems. Accordingly, deeper understanding of the phenotype and genotype of these bacteria would broaden our knowledge about the metabolic pathways that might play a crucial role in the biogeochemical cycling of elements on the early Earth, in the period of the Great Oxidation Event (2.4–1.8 Ga).

The aim of our work was to fill the knowledge gap on the metabolic pathways driving the respiration of sulfur and iron compounds in *D. alkaliphilus* strains. We also aimed to propose how the interrelations between the redox transformations of iron and sulfur compounds within a single species might affect its adaptation to geochemically contrasting extreme environments. To achieve our goals, we used a combination of classical microbiological methods with multi-omics analysis. We also collected the data on the geology of the Lake Magadi and the Fe-depleted subsurface Yessentuki aquifer to assess the significance of geochemical factors in the evolution of *D. alkaliphilus* energy metabolism.

## Materials and methods

### Strains and cultivation conditions

The strain AHT1^T^ isolated from a mixed anaerobic sediments of northeastern Mongolian soda lakes (Sorokin et al., 2008) was kindly provided for the study by Dr. D. Yu. Sorokin. The ability of strain AHT1^T^ for dissimilatory iron-reduction was tested in an optimal liquid medium of the following composition (per liter distilled water): NH_4_Cl 0.5 g; KH_2_PO_4_, 0.2 g; MgCl_2_ × 6H_2_O 0.1 g; CaCl_2_ × 2H_2_O 0.02 g; KCl 0.2 g; NaCl 3.0 g; Na_2_CO_3_ 15.0 g; NaHCO_3_ 20.0 g; Na_2_S × 9H_2_O 0.1 g; 1 mL trace element solution (Kevbrin and Zavarzin, 1992); 1 mL vitamin solution (Wolin et al., 1963); yeast extract 0.2 g. Molecular hydrogen (10% v/v in the gas phase) was used as the electron donor, and SF prepared as previously described (Zavarzina et al., 2006) was used as the electron acceptor. SF was added to the culture medium prior to sterilization to achieve a final Fe(III) content of 50 mM. The medium was prepared under pure N_2_ flow. Afterwards, NaHCO_3_, vitamins, Na_2_S × 9H_2_O, and SF were added. The medium was dispensed in 20 mL aliquots into 50 mL flasks and the headspace was filled with pure N_2_. The medium was autoclaved at 1 atm, 121°C for 20 min. The pH of the sterile medium was 9.5. After three successful transfers to the optimal medium, sodium sulfide and yeast extract were omitted for further cultivation steps in order to test the ability of strain AHT1^T^ to reduce SF lithoautotrophically in the absence of any reducing agents.

Strain Z-1002 was isolated in 2010 from the sediments of a hypersaline soda lake Magadi (Kenya). Anaerobic sediment samples were collected by Prof. G.A. Zavarzin in 1996 in pre-sterilized flasks, sealed on-site and stored since then at +4°C. The ability of strain Z-1002 to reduce SF during autotrophic growth with molecular hydrogen or formate in the presence of vitamin solution was previously reported (Zavarzina et al., 2018). Here we focused on determining its optimal growth conditions, the ability to reduce sulfur compounds and capacity for chemoorganotrophic growth.

The basal medium (BM) used for physiological tests of both strains contained (per liter of distilled water): NH_4_Cl 0.5 g; KH_2_PO_4_ 0.2 g; MgCl_2_ × 6H_2_O 0.1 g; CaCl_2_ × 2H_2_O 0.02 g; KCl 0.2 g; Na_2_S·9H_2_O 0.1 g, 1 mL trace elements solution (Kevbrin and Zavarzin, 1992), 1 mL vitamins solution (Wolin et al., 1963). Sodium formate (1 g l^−1^) and SF [final Fe(III) content of 50 mM] were added to test the Fe(III) reducing activity under optimal growth conditions. The pH growth range of strain Z-1002 was determined on BM medium supplemented with 60 g l^−1^ NaCl and 10 g L^−1^ NaHCO_3_. pH lower than 8.0 was adjusted with 6 M HCl, in the range between 8.0 and 9.5—by titrating with 10% Na_2_CO_3_ solution, in the range between 9.5 and 10.3—with 12 M NaOH solution. In this experiment, three tubes with culture media were prepared for each pH value: two tubes for cultivation and one for measuring the initial pH value of a medium after autoclaving. The рН stability of the cultures was checked at the end of the incubation.

The carbonate/bicarbonate growth optimum was determined on BM medium supplemented with 20 g L^−1^ NaCl, 55 g L^−1^ NaHCO_3_, and 95 g L^−1^ Na_2_CO_3_. This modified medium was gradually diluted with another one—BM containing 20 g L^−1^ NaCl but without carbonate/bicarbonate. In the latter medium, the optimal pH value for growth was maintained by the addition of 20 mM N-cyclohexyl-2-aminoethanesulfonic acid (CHES) buffer (Merck). Three subsequent transfers were performed to confirm the ability of strain Z-1002 to grow without carbonates.

The sodium chloride dependence of strain Z-1002 was determined on BM medium supplemented with 20 g L^−1^ NaHCO_3_ and 40 g L^−1^ Na_2_CO_3_. NaCl was added to this medium at the concentrations of (g L^−1^): 0, 3, 5, 10, and up to 140 in increments of 10. Three subsequent transfers were performed to confirm the ability of strain Z-1002 to grow without chloride.

In all the experiments, soluble electron donors and acceptors were added from sterile anaerobic stock solutions prior to inoculation. All the organic substrates (peptides, carbohydrates, alcohols and organic acids) were filter-sterilized using 0.2 μm pore size syringe filters (Millipore) and added to a final concentration of 3 g L^−1^. The capability for dissimilatory reduction of sulfur compounds (S^0^ (1% w/v); S_2_O_3_^2−^ (10 mM); SO_4_^2−^ (20 mM)) was tested with formate (1 g L^−1^), H_2_ (10% v/v in the gas phase), ethanol or organic acids (10 mM in each case) added as the electron donors. The ability to disproportionate sulfur or thiosulfate was tested without the addition of any of the electron donors.

The ability to oxidize a natural siderite-based mineral mixture (Fe_2_CO_3_ as the major component) under anaerobic conditions was tested using an optimized medium without sodium sulfide with the following composition (per liter distilled water): NH_4_Cl 0.5 g; KH_2_PO_4_ 0.2 g; MgCl_2_ × 6H_2_O 0.1 g; CaCl_2_ × 2H_2_O 0.02 g; KCl 0.2 g; NaCl 50.0 g; Na_2_CO_3_ 40.0 g; NaHCO_3_ 20.0 g; 1 mL trace mineral solution (Kevbrin and Zavarzin, 1992); 1 mL vitamin solution (Wolin et al., 1963). This medium was dispensed under N_2_ flow in 10 mL aliquots into Hungate tubes containing 0.1 g dry mineral powder each. Uniform grains of the siderite-based mineral mixture of hydrothermal origin (Bakal deposit, the Urals, Russia) were preliminarily selected and ground to powder (<100 μm particle size) in an agate mortar. The presence of the Fe(III)-containing green rust (10.6%) and iron oxides (13%) in the mineral mixture was detected by Mössbauer spectroscopy (Table 1). The siderite-containing medium was autoclaved at 1 atm, 121°C for 20 min. Three subsequent transfers were performed to confirm the ability of strain Z-1002 to grow with siderite by Fe(II) oxidation.

**Table 1:** Hyperfine parameters of room temperature ^57^Fe Mössbauer spectra of the siderite-based mineral mixture incubated with the growing culture of strain Z-1002 and in a sterile control medium.

Subspectrum										Phase	*I*, %	δ, mm/s	ε, mm/s	Г, mm/s				
Siderite-based mixture from uninoculated control
D_1_	Siderite	76.3 ± 2.0	1.229 ± 0.001	0.884 ± 0.001	0.241 ± 0.002				
D_2_	Green rust (Fe^2+^)	8.4 ± 2.1	1.17 ± 0.03	1.02 ± 0.05	0.64 + 0.09				
D_3_	Green rust (Fe^3+^)	2.2 ± 0.5	0.37 ± 0.04	0.34 ± 0.03	0.39 ± 0.06				
		*I*, %	δ_max_, mm/s	ε_max_, mm/s	*B*_max_, T	δ_av_, mm/s	ε _av_, mm/s	*B*_av_, T	Г, mm/s
S	Iron oxides	13.0 ± 0.4	0.379 ± 0.016	−0.092 ± 0.017	49.0 ± 0.2	0.385 ± 0.012	−0.073 ± 0.011	46.0 ± 0.3	0.21 ± 0.06
Siderite-based mixture from the grown culture of strain Z-1002									
Subspectrum	Phase	*I*, %	δ, mm/s	ε, mm/s	Г, mm/s				
D_1_	Siderite	76.1 ± 0.7	1.228 ± 0.001	0.883 ± 0.003	0.267 ± 0.001				
D_2_	Green rust (Fe^2+^)	2.1 ± 0.6	1.226 ± 0.130	1.209 ± 0.120	0.43 ± 0.09				
D_3_	Green rust (Fe^3+^)	4.6 ± 0.4	0.36 ± 0.08	0.34 ± 0.09	0.50 ± 0.05				
		*I*, %	δ_max_, mm/s	ε_max_, mm/s	*B*_max_, T	δ_av_, mm/s	ε _av_, mm/s	*B*_av_, T	Г, mm/s
S	Iron oxides	17.2 ± 0.4	0.369 ± 0.012	−0.097 ± 0.012	49.3 ± 0.1	0.374 ± 0.008	−0.092 ± 0.008	46.5 ± 0.2	0.23 ± 0.03

I, relative intensity of a subspectrum; δ, Mössbauer line shift relatively α-Fe; ε, quadrupole shift; Г, line width; δ_max_, δ_av_, maximum and average values of Mössbauer line shift; ε_max_, ε_av_, maximum and average values of a quadrupole shift; B_max_, B_av_, maximum and average values of hyperfine magnetic field.

All the cultivation experiments were performed in duplicate using Hungate tubes.

### Sampling of Yessentuki mineral water and enrichment of a novel *Dethiobacter* phylotype

Subsurface water from the Yessentuki Mineral Water Deposit (YMWD, Stavropol Krai, Russia) was sampled in September, 2020, from Well 9 extracting Na-Ca-HCO_3_-SO_4_-type mineral water from the Lower Cretaceous (K_2_*s*-*m*) aquifer. This well (E 42°48′10” N 44°2′30″) is 600 m deep and has open boreholes in the interval of 485–556 m. The temperature, pH value, and salinity of the mineral water at the wellhead during the sampling were 21.9°C, 7.9, and 0.4 g L^−1^, respectively. One hundred liter of water was filtered through a 0.2 μm pore size track membrane filters (JINR, Dubna, Russia) under the natural overpressure of the well, as described previously (Gavrilov et al., 2022). The filter was cut in half and one of its parts was used for DNA extraction and phylogenetic profiling of the natural water microbial community according to a previously described procedure (Gavrilov et al., 2022). Another part of the filter was placed in a pre-sterilized 100 mL flask, filled with mineral water (80 mL) and CO_2_ gas headspace (20 mL), and was stored at +4°C for 1.5-years. After storage, the flask was supplemented with SF [final Fe(III) content of 10 mM], sodium sulfate, sodium acetate, and sodium formate (10 mM each), and used as a primary enrichment of sulfate and iron reducing microorganisms inhabiting the mineral water. It was incubated in the dark at 35°C for 3 weeks. Subsamples for DNA extraction and phylogenetic profiling were taken from this culture at the beginning and after the end of the incubation. Further transfers from this primary enrichment were made to the medium with the following composition (per liter distilled water): NH_4_Cl 0.015 g; KH_2_PO_4_ 0.115 g; MgCl_2_ × 6H_2_O 0.6 g; CaCl_2_ × 2H_2_O 0.1 g; NaHCO_3_ 0.3 g; Na_2_S·9H_2_O 0.1 g, 1 mL trace element solution (Kevbrin and Zavarzin, 1992); 1 mL vitamin solution (Wolin et al., 1963). Sodium formate (1 g L^−1^) or sodium acetate (1 g L^−1^) were used as the electron donors and SF (final Fe(III) content of 50 mM) as the electron acceptor. The medium for the enrichments was prepared by boiling and cooling it under pure N_2_ flow, then NaHCO_3_, vitamins, Na_2_S·9H_2_O, and SF were added, and the pH was adjusted to 8.0 with 2.5 M NaOH solution. The medium was dispensed in 10 mL aliquots into 16 mL Hungate tubes and autoclaved at 1 atm, 121°C for 20 min.

### Phenotypic characterization of the strains

Growth of *D. alkaliphilus* strains was monitored by direct cell counting using an Axio Lab.A1 phase-contrast and fluorescent microscope (Zeiss, Germany). Subsamples of SF-grown cultures were pre-stained with acridine orange dye for DNA.

Molecular hydrogen consumption, the formation of gaseous metabolites, sulfide concentration, and Fe(II) production were monitored as previously described (Khomyakova et al., 2022). Acetate was analyzed using the same chromatograph with FID detector and an Optima FFAPplus 0.25 μm × 0.32 mm × 30 m capillary column (Macherey-Nagel) with argon as the carrier gas. Separation was carried out with temperature programming. Samples for gas–liquid chromatography (0.2 mL each) were pre-treated by centrifugation at 12,600 g for 2 min, followed by stepwise acidification of the clear supernatants with H_3_PO_4_ and 5 M formic acid to the pH of 2.0. The detection limit of the method was 0.2 mM.

### Mössbauer spectroscopy

The Fe^2+^/Fe^3+^ ratio of minerals was determined by ^57^Fe Mössbauer spectroscopy. This method allows for the determination and quantification of different atomic environments, magnetic states, chemical states and transformations of iron-containing compounds (Kamnev and Tugarova, 2021). All Mössbauer measurements were performed at room temperature using the MS-1101 Em spectrometer, operating in the constant acceleration mode, with a ^57^Co source in the Rh matrix. The calibration was carried out relating to α-Fe, and the spectra were fitted using SpectrRelax software. To process the spectra, a model fitting was carried out simultaneously with the extraction of the hyperfine magnetic field distribution.

### DNA extraction and amplicon sequencing

The composition of the microbial community of Yessentuki mineral water and of the enrichments was determined by amplification of the hypervariable V4 region of the 16S rRNA genes, followed by sequencing and bioinformatic data processing. Total DNA was isolated with the FastDNA^™^ Spin Kit for Soil DNA Extraction (MP Biomedicals, United States) according to the manufacturer’s instructions using the FastPrep-24™ 5G Bead Beating System (MP Bio, United States). Amplicon libraries were prepared as described by Gohl et al. (2016) using the following primers: 515F (5′-GTG BCA GCM GCC GCG GTA A-3′) and 806R (5′-GAC TAC NVG GGT MTC TAA TCC-3′) (Hugerth et al., 2014; Merkel et al., 2019) including Illumina technical sequences (Fadrosh et al., 2014). High-throughput sequencing of the libraries was performed using MiSeq Reagent Micro Kit v2 (300-cycles) MS-103-1,002 (Illumina, United States) on a MiSeq sequencer (Illumina, United States) according to the manufacturer’s instructions. Libraries were prepared and sequenced in two replicates for each sample. Amplicon sequence variants (ASV) were obtained using the Dada2 script (Callahan et al., 2016) and the SILVA 138.1 database (Quast et al., 2013). Analysis of the ASV tables was performed using Rhea (Lagkouvardos et al., 2017). All sequencing data were deposited in SRA (NCBI) under BioProject ID PRJNA945437.

### Genome sequencing and analysis

A WGS library preparation and sequencing of *Dethiobacter alkaliphilus* Z-1002 was performed in BioSpark Ltd., Moscow, Russia, using KAPA HyperPlus Library Preparation Kit (KAPA Biosystems, United Kingdom) according to the manufacturer’s protocol and NovaSeq 6,000 system (Illumina, San Diego, CA, United States) with a reagent kit capable of read 100 nucleotides from each end. This Whole Genome Shotgun project has been deposited at DDBJ/ENA/GenBank under the accession JAPDNO000000000. Gene search, annotation, and genome-based phylogenetic reconstructions were performed as previously described (Khomyakova et al., 2022) with an additional use of PGAP service (Li et al., 2021).

Screening of the genomes of both *D. alkaliphilus* strains for multiheme cytochromes and their sequence analysis was performed as previously described (Toshchakov et al., 2018) using reported cytochromes, involved in extracellular electron transfer (EET) in *Geobacter sulfurreducens*, *Shewanella oneidensis*, “*Thermincola potens*,” *Carboxydothermus ferrireducens* (Gavrilov et al., 2021 and references therein), the dataset of cytochrome query sequences was supplemented with multiheme proteins reported to be involved in Fe(III) respiration or other EET processes in the thermophilic Fe(III) reducers *Carboxydocella thermautotrophica* (Toshchakov et al., 2018) and *Melioribacter roseus* (Gavrilov et al., 2017), anaerobic Fe(II) oxidizing bacteria *Sideroxydans lithotrophicus*, *Gallionella capsiferriformans*, and *Dechloromonas aromatica* (Chakraborty et al., 2005; Emerson et al., 2007; Liu et al., 2012), Fe(III) reducing and syntrophic archaea (Mardanov et al., 2015; Smith et al., 2015; Krukenberg et al., 2018; Kashyap and Holden, 2021). Heme-binding motifs in *D. alkaliphilus* multihemes were predicted as previously described (Mardanov et al., 2015). Conservative domains, transmembrane helices and signal peptides were predicted using the hmmscan^1^ web-service (Potter et al., 2018) with default parameters and all databases included.

### Phylogenetic analysis of cytochromes

The cytochrome *c* protein sequences of SirA octaheme sulfite reductase from *S. oneidensis* and the MtoA Fe(II)-oxidizing decaheme of *D. aromatica* were retrieved from the non-redundant NCBI protein database on November 2022. Homologs for each of these sequences were separately screened for using the BLASTp algorithm and the obtained sets of sequences were manually curated and processed as described previously (Gavrilov et al., 2021). The resulting sets of 48 amino acid sequences for the SirA query and 342 sequences for MtoA query were amended with the sequences of SirA, or the MtoA homologs from the genomes of *D. alkaliphilus* strains (Supplementary Table S1). The immunoglobulin-like domains were omitted from the sequences of *D. alkaliphilus* to decrease non-specific alignments within the protein sets. The sets were then aligned using MAFFT 7.490 with default parameters, with 1,000 iterations of the FFT-NS-i refinement method (Katoh et al., 2019). Two final alignments (for SirA and MtoA hits) were subjected to a Bayesian inference and used to construct unrooted phylogenetic consensus trees as described previously (Gavrilov et al., 2021).

### Shotgun proteomic analysis

For proteomic analysis, biomass of *D. alkaliphilus* strain AHT1^T^ grown with H_2_ and thiosulfate or SF as the electron acceptors was harvested from 100 mL cultures by centrifugation at 16,000 g for 15 min. Both cultures were grown in triplicate. Before harvesting, the SF-grown culture was separated into liquid and mineral phases using low speed centrifugation (5 min, 1,000 g). The mineral phase was ultrasonicated inside the centrifuge bags in an ultrasonic bath (Sapphire, Russia) at 99% power, 5 min, to separate adherent cells from the minerals. The ultrasonicated mineral phase was then combined with the liquid phase, mixed thoroughly, the bulk of the minerals were held in the bags with a hand magnet and the remaining suspension was transferred to another centrifuge bag to harvest the biomass at 13,000 g for 20 min.

Cell lysis, reduction, alkylation and digestion of the proteins, as well as the processing and LC-MS/MS analysis of the obtained peptide sets were performed as previously described (Gavrilov et al., 2021). Label-free protein quantification was made by MaxQuant software version 1.5.6.5 using *D. alkaliphilus* strain AHT^1^ amino acid FASTA dataset and a common contaminant database through the Andromeda search engine according to the previously described protocol (Gavrilov et al., 2021). The iBAQ algorithm (Schwanhäusser et al., 2011) implemented in MaxQuant software was used to quantify proteins in each sample. Normalization of each protein’s iBAQ value to the sum of all iBAQ values generated a relative iBAQ (riBAQ) values corresponding to the molar percentage of each protein in the sample, with the entire set of proteins in the sample taken as 100% (Shin et al., 2013).

The mass spectrometry proteomics data were deposited at the ProteomeXchange Consortium via the PRIDE (Perez-Riverol et al., 2022) partner repository with the dataset identifier PXD040929.

### Analysis of environmental distribution of *Dethiobacter* genus and *Dethiobacteraceae* family

We used 16S rRNA gene sequences and related metadata to analyze the relative abundance of *Dethiobacters* in different environments. For this purpose, we took all sequences assigned to the *Dethiobacter* genus in the SILVA database 138.1 (Quast et al., 2013). Next, we analyzed the similarity of these sequences to the 16S rRNA gene sequences of *D. alkaliphilus* strains AHT1^T^ and Z-1002 using BLASTN (Altschul et al., 1990), discarding all sequences with less than 94.5% similarity to these cultured strains of the genus. Finally, to analyze the distribution of the genus *Dethiobacter* in different types of ecotopes, we obtained 108 sequences of 16S rRNA genes. Next, we analyzed the metadata related to the detection sources of these sequences.

To analyze the distribution of the representatives of the *Dethiobacteraceae* family (Sorokin and Merkel, 2019), we used the metadata on MAGs from the GTDB 207 database (Parks et al., 2022), i.e., the information on isolation sources, description of sampling sites, geological or geochemical information on them if publicly available.

## Results

### General phenotypic characteristics of strain Z-1002

The Fe(III)-reducing strain of *D. alkaliphilus*, Z-1002, was previously isolated in a pure culture with SF and formate under highly alkaline conditions (Zavarzina et al., 2018). Here, we describe its metabolic characteristics in detail. Strain Z-1002 appeared to be an obligate alkaliphile with the pH growth range from 7.8 to 10.1 and an optimum at pH 9.2. It is an obligate natronophile growing in the range of carbonate/bicarbonate concentration ratios from 13.5/7.5 to 82.0/45.0 g L^−1^ with an optimum at 40.0/20.0 g L^−1^. It could not grow without carbonates in the presence of CHES buffer after the second transfer from a carbonaceous medium. It was halotolerant and grew in the range of NaCl concentrations from 0 to 120 g L^−1^ with an optimum at 50–60 g L^−1^. Strain Z-1002 grew chemoorganotrophically with lactate, succinate, butyrate, pyruvate, propionate, or ethanol as the electron donors and SF or thiosulfate as the electron acceptors. Slow growth with a low cell yield of 5 × 10^5^ cells mL^−1^, accompanied by the production of 2.5 mM sulfide, was observed on thiosulfate with the addition of formate or H_2_ as electron donor. The most intense thiosulfate reduction accompanied by the formation of 15 mM sulfide was observed with ethanol as the electron donor. Strain Z-1002 was unable to use elemental sulfur and sulfate as the electron acceptors, as well as it was unable to disproportionate sulfur or thiosulfate. Strain Z-1002 was unable to grow by fermentation of carbohydrates, peptides, and amino acids.

### Fe(III) reducing ability of the type strain AHT1^T^

**Figure 1:**
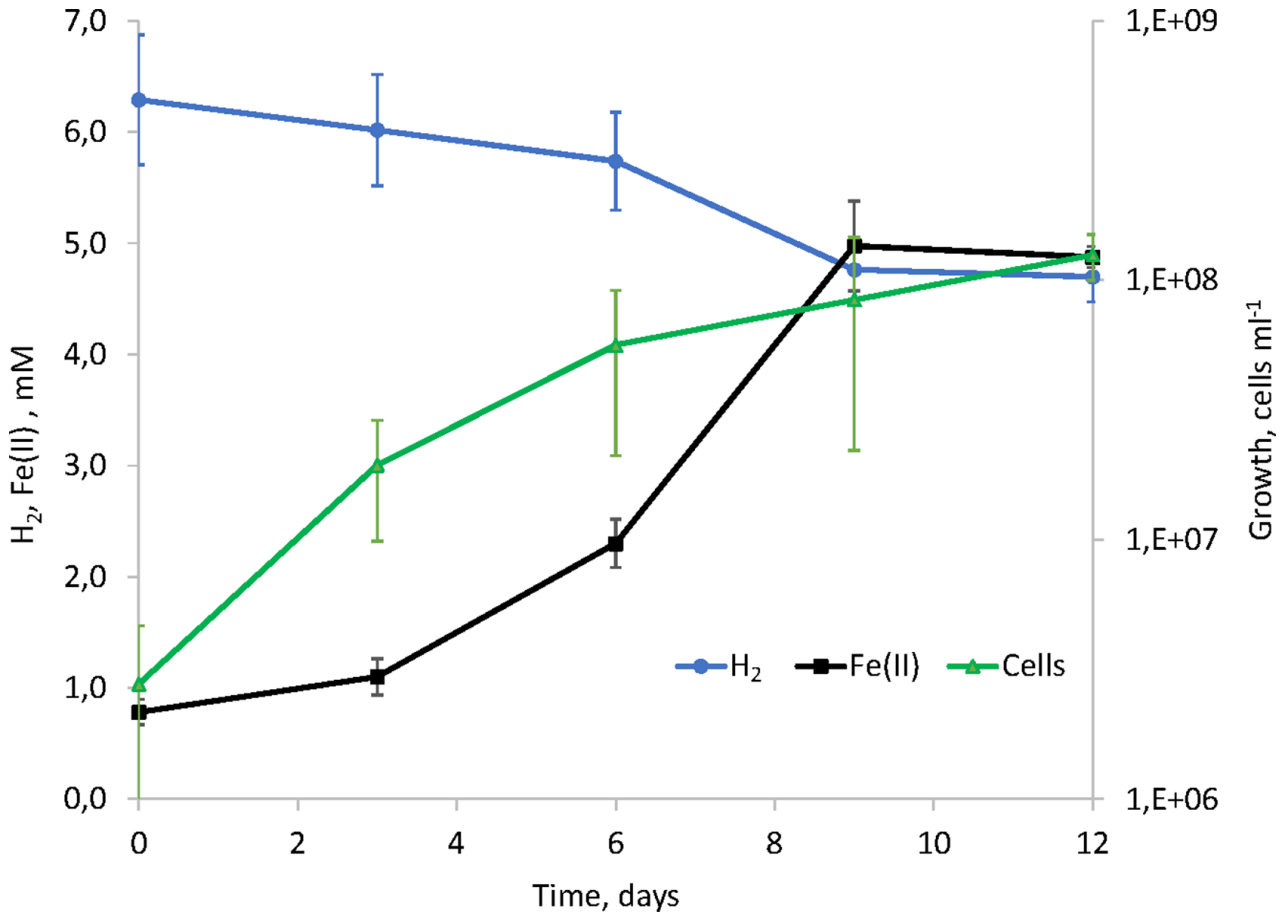
Kinetics of hydrogenotrophic growth of strain AHT1^T^ with ferrihydrite as the electron acceptor, hydrogen consumption and Fe(II) production in the absence of yeast extract and sodium sulfide.

**Figure 2:**
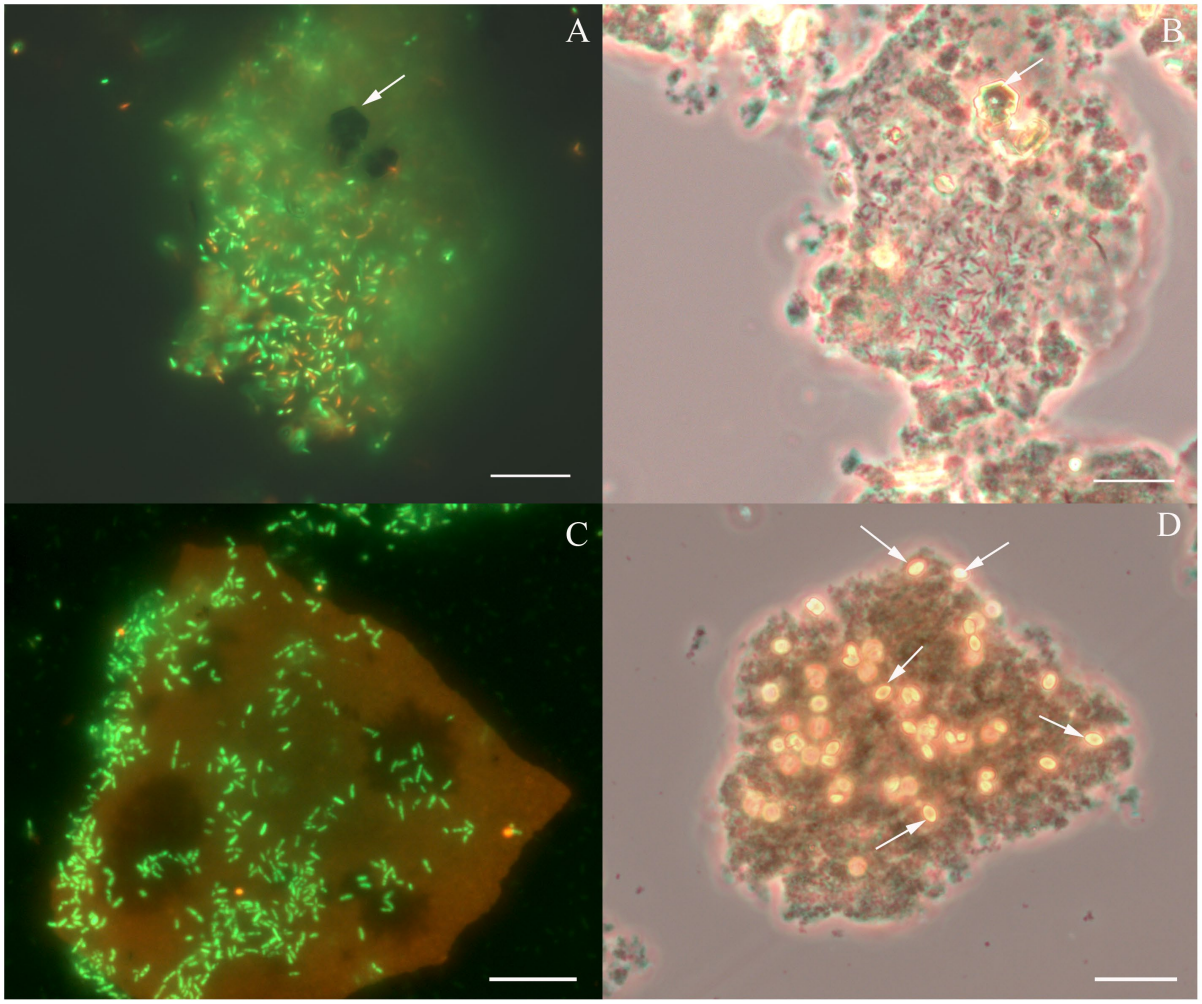
Cellular morphology of *Dethiobacter alkaliphilus* strains. **(A)** Fluorescent micrograph of acridine orange stained culture of strain Z-1002 colonizing a synthesized ferrihydrite particle during the growth with molecular hydrogen, *white arrow* indicates a newly formed magnetite crystal. **(B)** Phase contrast micrograph of the same culture (same spot), *white arrow* indicates a newly formed magnetite crystal. **(C)** Fluorescent micrograph of acridine orange stained culture of strain AHT1^T^ colonizing a synthesized ferrihydrite particle during the growth with molecular hydrogen. **(D)** Phase contrast micrograph of the same culture and the same spot, *white arrows* indicate newly formed siderite crystal. Each bar = 10 μm.

Considering the metabolic features of *D. alkaliphilus* isolate from the lake Magadi, we tested the ability of the type strain AHT1^T^ for dissimilatory iron reduction. After three sequential transfers on the medium supplemented with SF and H_2_ in the absence of yeast extract and sodium sulfide, strain AHT1^T^ produced 4.1 ± 0.5 mM Fe(II) from ferrihydrite with concomitant oxidation of 1.6 ± 0.2 mM molecular hydrogen (Figure 1). The mineral phase changed from brownish to dark brown color during the growth, indicating the formation of Fe(II)-containing minerals (Supplementary Figures S1). The cell yield under these conditions was 3.5 ± 2 × 10^7^ cell mL^−1^. Ferrihydrite-grown cells of both *D. alkaliphilus* strains were strongly associated with iron minerals (Figure 2).

### Experimental verification of the ability of strain Z-1002 for anaerobic Fe(II) oxidation

Cultures of the strain Z-1002 appeared to oxidize Fe(II) from a natural mixture of hydrothermal siderite (76%), green rust (11%), and iron oxides (13%) under anaerobic conditions in the absence of any organic compounds. The maximum cell yield under these growth conditions was 10^7^ cells mL^−1^ and was observed by the 34th hour of incubation (Supplementary Figure S2). Growth was accompanied by the changes in the mineral phase as no soluble Fe(III) or Fe(II) species were detected in the culture during the incubation. Mössbauer analysis of the minerals from the grown cultures (stationary growth phase) and uninoculated controls revealed a decrease in the relative total intensity of the spectra corresponding to ferrous iron atoms from 87.7 ± 2.9% to 78.2 ± 0.9% in the grown cultures (Table 1). No such changes were observed in the controls. The relative intensity of siderite subspectrum of the microbially impacted mineral sample remained virtually unchanged, but the intensity of the doublet corresponding to Fe^2+^ atoms in the green rust structure decreased from 8.4 ± 2.1% to 2.1 ± 0.6%, while the intensity of the doublet corresponding to Fe^3+^ atoms in this phase increased from 2.2 ± 0.5% to 4.6 ± 0.4% (Table 1 and Supplementary Figure S3). This means that 75% of the bivalent iron atoms contained in the green rust were oxidized. At the end of the incubation, we also observed an increase of 4.2% in the magnetically ordered phase content of the mineral mixture. Thus, on the one hand, we have observed a rearrangement of the bivalent and trivalent iron atoms in the green rust, and on the other hand, an increase in the amount of iron oxides in the mixture.

### Genome analysis of *Dethiobacter alkaliphilus* strains

#### Genomes statistics

The genome of the type strain AHT1^T^ was previously sequenced and annotated (Melton et al., 2017). Here we have sequenced and analyzed the genome of strain Z-1002 and compared its genomic determinants of central carbon and energy metabolism with the type strain, as well as clarified the phylogenomic position of the genus *Dethiobacter*. Detailed statistics of both *D. alkaliphilus* genomes can be found in Supplementary Table S2.

#### Phylogenomic position of the genus *Dethiobacter*

**Figure 3:**
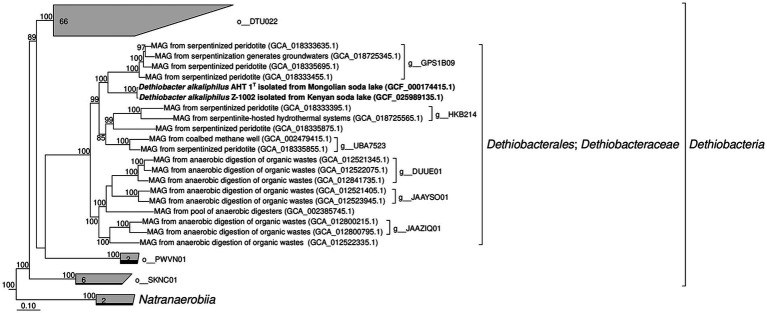
Phylogenomic placement of *Dethiobacter alkaliphilus* strains and MAGs of the *Dethiobacteraceae* family based on concatenated partial amino acid sequences of 120 bacterial single copy conserved marker genes with taxonomic designations according to the GTDB (Release 07-RS207). Bootstrap consensus tree is shown with values above 85% placed at the nodes. Bar, 0.1 changes per position.

Initially, the description of the phylogenetic position of the genus *Dethiobacter* was based only on the analysis of the 16S rRNA gene, which showed that this genus represents a deep phylogenetic lineage within the *Firmicutes* (*Bacillota*) phylum. Based on a modern phylogenomic reconstruction approach using 120 single-copy conserved marker genes (Parks et al., 2022), *D. alkaliphilus* has been classified as an individual class of “*Dethiobacteria*” (Sorokin and Merkel, 2022). Apart from *D. alkaliphilus*, this class includes only metagenome-assembled genomes (MAGs). According to the results of our phylogenomic analysis based on the same method, the MAGs of the *Dethiobacteraceae* family appeared to form two distinct phylogenetic clusters: one containing *D. alkaliphilus* and the MAGs from serpentinizing environments, and another containing MAGs from anaerobic digestion facilities and organic wastes (Figure 3).

#### Central metabolism of *Dethiobacter alkaliphilus*

As expected, both strains of *D. alkaliphilus* possess similar gene sets determining central metabolic processes. These are identical clusters of genes encoding [NiFe] uptake hydrogenase (HydABC) and its assembly proteins HypDEF, complete gene sets of V-type Na^+^-ATP synthetase, and the Wood–Ljungdahl pathway for autotrophic CO_2_ fixation, which includes an operon comprising a CODH gene *cooS* followed by *acsABCDE* genes of acetyl-CoA-synthetase and separately located genes encoding the methylTHF branch of the pathway and a formate dehydrogenase. Interestingly, both strains possess duplicates of the *cooS* genes, which are encoded separately by the CODH operons (DealDRAFT_0192 in strain AHT1^T^ and OMD50_RS09960 in Z-1002 strain). No genes of energy-converting hydrogenases have been identified in *D. alkaliphilus*. The genomes of both strains encode electrogenic membrane Na^+^/H^+^ Mrp antiporters, which are essential for natronophiles to expel sodium from the cell. Also, both genomes contain similar gene sets determining the processes of osmotic balancing, namely those, encoding sucrose-phosphate phosphatases and sucrose synthases, as well as ABC transporters specific for betaine and choline. Oxidation of short chain organic acids is determined in both strains by highly similar genes of tetrameric pyruvate oxidoreductases, succinate dehydrogenases/fumarate reductases (OMD50_RS06125-30 and DealDRAFT_0594–96 loci, respectively), and acetyl-CoA-synthetases encoded adjacently to CODH subunits.

#### Reduction and oxidation of iron minerals

**Figure 4:**
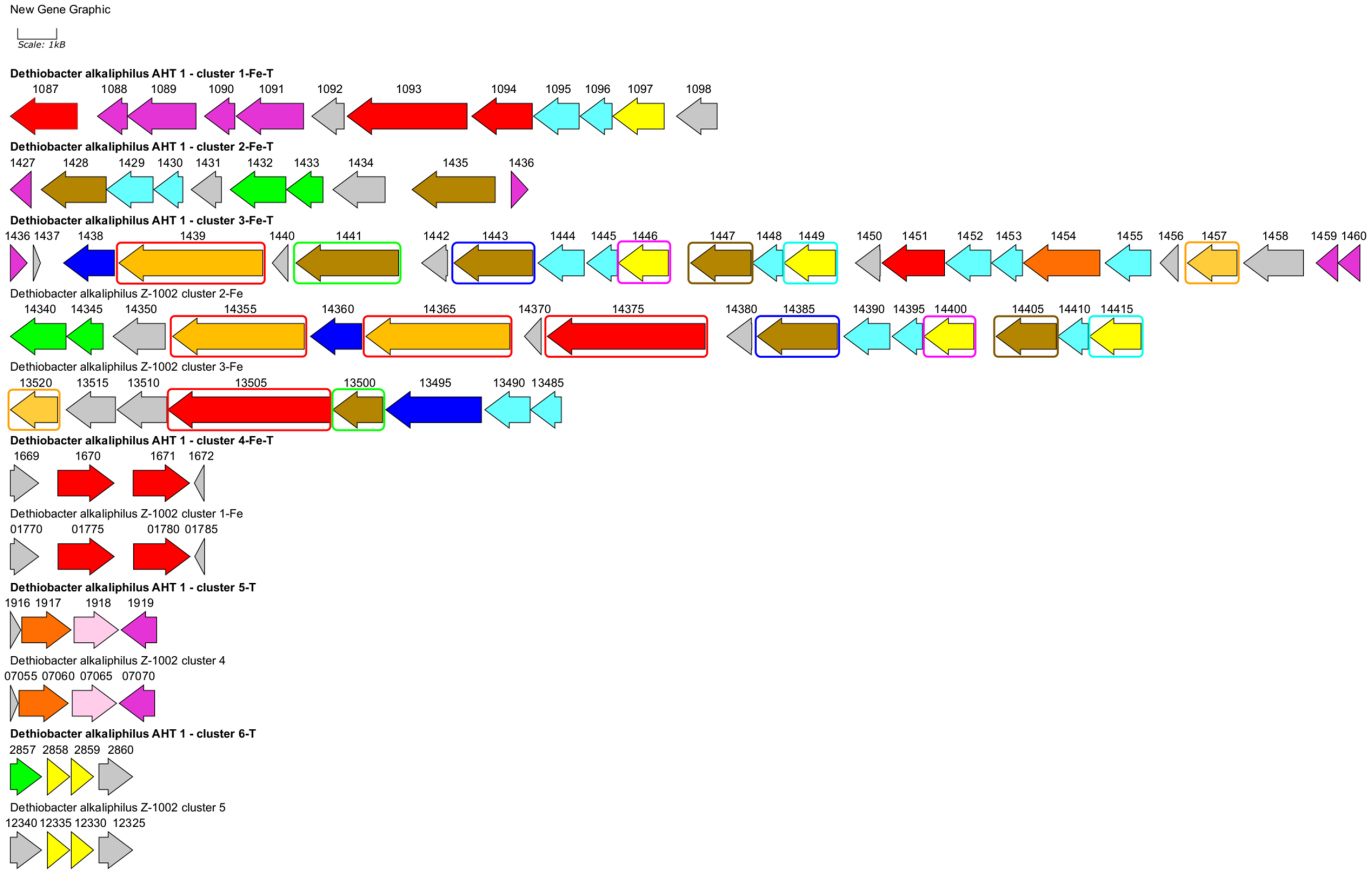
Clusters of EET-related genes and their close genomic neighborhood in *D. alkaliphilus* strains AHT1^T^ (marked with *bold* text) and Z-1002. Clusters are numbered according to their genomic coordinates. Gene mapping is performed with Gene Graphics web application (Harrison et al., 2018). Genes are marked with the numbers of corresponding locus tags with their prefixes omitted (DealDRAFT_ for the strain AHT1^T^ and OMD50_RS for the strain Z-1002). Genes are colored according to their predicted products: *green*—flavoproteins, *blue*—transport system proteins, *light blue*—NHL- and TPR-repeat-containing proteins, *pink*—rhodanese domain-containing proteins, *red*—homologs of putative terminal Fe(III) reductases, *olive*—homologs of various EET-related secreted multihemes, *light brown*—homologs of MtoA-type Fe(II) oxidases, *dark orange*—homologs of octaheme SirA sulfite reductases and Otr tetrathionate reductases, *yellow*—putative quinol oxidases, *gray*—other proteins including hypothetical ones. Similar genes of multiheme cytochromes in the clusters 3-Fe-T, 2-Fe, and 3-Fe are marked with differentially colored rectangles. Clusters 4-Fe-T and 1-Fe, 5-T and 4, as well as 6-T and 5 are almost identical to each other, that is not specially marked on the scheme. Homology values for all the mentioned similar genes are given in Supplementary Table S2.

Strain AHT1^T^ has been previously reported to possess a wide repertoire of multiheme *c*-type cytochromes including those thought to be involved in nitrate reduction, redox transformation of sulfur and Fe(III) compounds (Melton et al., 2017; Zavarzina et al., 2018; Sorokin and Merkel, 2019). Here we have revisited previous annotations of multiheme genes predicted in AHT1^T^ and compared them with cytochrome-encoding genes from strain Z-1002. A comprehensive analysis revealed 31 proteins with *c*-type multiheme conserved domains encoded in the AHT1^T^ genome and 27 multiheme proteins encoded in the Z-1002 genome. All the multihemes of the latter strain share homology with those of the type strain (31–100% amino acid sequence identity, Supplementary Table S1), whereas the AHT1^T^ genome encodes 7 multihemes that have no homology to the proteins of the strain Z-1002. The majority of multihemes from both *D. alkaliphilus* strains share similarity with the proteins previously reported to be involved in EET to Fe(III) minerals in *Geobacter*, *Shewanella*, *Thermincola*, *Carboxydothermus* species, or in Fe(III)-reducing archaea (Supplementary Table S1). In addition, 2 multihemes from AHT1^T^ and 3 multihemes from Z-1002 share similarities (22–27% identity) with MtoA-type outer surface cytochromes that perform the reverse EET process of Fe(II) oxidation within MtoABC porin-cytochrome complexes (Shi et al., 2012). The majority of EET-related multihemes, whether putative Fe(III) reductases or Fe(II) oxidases, are encoded by clustered genes in both strains of *D. alkaliphilus*. However, the structure of these gene clusters differs in the two strains (Figure 4). In total, 6 such clusters were identified and numbered according to their genomic coordinates in strain AHT1^T^ and 5—in strain Z-1002. The clusters determining the EET processes are likely to encode not only the terminal oxidoreductases, interacting with extracellular electron acceptors or donors, but also the proteins linking these oxidoreductases with the membrane-bound electron transfer chain, quinol oxidizing cytochromes and auxiliary proteins involved in the secretion and proper spatial localization of the components of the EET chain within the cell envelope and on the cell surface (Shi et al., 2012). The strain AHT1^T^ possesses four such clusters. The cluster 1-Fe-T (of the type strain AHT1^T^) contains 3 multiheme genes homologous to those of SmhB cytochrome (38% identity) specific for soluble Fe(III) complexes in *C. ferrireducens* (Gavrilov et al., 2021), the key multiheme MtrA (22% identity) of the EET pathway in *S. oneidensis* which transfers electrons across the outer membrane to extracellular acceptors (Campbell et al., 2022), and a homolog of a quinol-oxidizing multiheme CymA (34% identity) that initiates the metal-reducing pathway in *S. oneidensis* (Shi et al., 2016). Cluster 1-Fe-T also encodes NHL- and TPR-repeat-containing proteins and includes a regulatory region downstream of the cytochrome genes. All the proteins encoded within this cluster have no homologs in the genome of strain Z-1002. Cluster 2-Fe-T in strain AHT1^T^ encodes two homologs (25 and 30% identity) of secreted *c*-type cytochromes from Gram-positive thermophilic Fe(III)-reducers “*T. potens*” and *C. ferrireducens* together with NHL- and TPR-repeat-containing proteins and electron transfer flavoproteins. The cluster is flanked with two regulatory genes (Figure 4). Only one protein from this cluster, DealDRAFT_1428, has a homolog in Z-1002 strain (Supplementary Table S1). The largest cluster 3-Fe-T of AHT1^T^, which was previously mispredicted to include the locus DealDRAFT_1428–35 (Zavarzina et al., 2018), encodes nine multiheme cytochromes. Two of these proteins (namely, DealDRAFT_1439 and 1457) are homologous (22 and 27% identity) to MtoA-type cytochromes, while the others are similar to CymA and various auxiliary multihemes of EET pathways in *C. ferrireducens* and an ANME-2 group archaeon (Gavrilov et al., 2021; Kashyap and Holden, 2021). Interestingly, the MtoA homologs of strain AHT1^T^ share varying similarity (from complete to 37% identity) with six different multihemes of strain Z-1002 (OMD50_RS14355, 14365, 14375, 09855, 13505, 13520, Supplementary Table S1). In total, seven multihemes of the 3-Fe-T cluster have homologs in strain Z-1002 (Figure 4). In addition to cytochromes, the 3-Fe-T cluster of strain AHT1^T^ contains the genes of several NHL- and TPR-repeat-containing proteins and a component of an ABC-type transport system.

In strain Z-1002, the majority of EET-related genes are organized in two clusters (Figure 4). The largest cluster 2-Fe encodes three homologs of the DealDRAFT_1,439 protein mentioned above (38–87% identity, Supplementary Table S1). Two of these homologs share similarity with the MtoA Fe(II)-oxidizing cytochrome (23 and 29% identity), and one—with phylogenetically related MtrD Fe(III)-reducing cytochrome (24% identity). Other multihemes encoded within the 2-Fe cluster of strain Z-1002 share homology with the CymA quinol-oxidizing cytochrome (35% identity) and auxiliary EET-related proteins of *C. ferrireducens* and *S. oneidensis* (29–31% identity). In addition, this cluster encodes electron transfer-related flavoproteins, NHL- and TPR-repeat-containing proteins, and an ABC transporter all of which might be involved to the secretion and proper orientation of EET-driving multihemes on the cell surface. Another large cluster of EET-related multiheme genes in strain Z-1002 is 3-Fe which also encodes an MtoA homolog (27% identity) OMD50_RS13520, completely identical to DealDRAFT_1,457, as well as the homologs (26–28% identity) of the periplasmic DmsE and outer cell surface OmcX cytochromes involved in the EET pathways of *S. oneidensis* and *G. sulfurreducens*, respectively.

Both strains of *D. alkaliphilus* possess three more EET-related gene clusters identical to each other (Figure 4). These small clusters encode the homologs of putative Fe(III) reducing multihemes (31–33% identity, clusters 4-Fe-T and 1-Fe), putative quinol-oxidizing cytochromes (29–32% identity, clusters 6-T and 5), and the cytochromes homologous to SirA/MccA-like sulfite reductases and rhodanese domain proteins (clusters 5-T and 4, see below).

Considering a rare case of the appearance of both Fe(III) reduction, and Fe(II) oxidation determinants in a single organism, we have analyzed the homologs of MtoA proteins from *D. alkaliphilus* strains in more detail. The protein DealDRAFT_1439 and all its four homologs from strain Z-1002 appeared to possess large immunoglobulin-like conserved domains at their C-terminal regions. The same domain was identified in the protein OMD50_RS09855 homologous to the MtrD Fe(III)-reducing cytochrome of *S. oneidensis* (22% identity), as well as to DealDRAFT_1457 MtoA-like protein which only possesses multiheme conserved domains (29% identity, Supplementary Table S1). Phylogenetic reconstruction of the multiheme domains of all the MtoA-like proteins from both *D. alkaliphilus* strains (DealDRAFT_1439, 1454, 1457, and OMD50_RS14355, 14365, 13520), together with related MtrA/D homologs from this species (DealDRAFT_1094, OMD50_RS14375, 09855), revealed separate clustering and deep phylogenetic branching of all *D. alkaliphilus* proteins from previously described DmsE-family decahemes involved in Fe(II) oxidation or Fe(III) reduction (MtoA proteins or MtrA/D proteins, respectively, Supplementary Figure S4). The protein DealDRAFT_1,439 clustered with its three homologs (OMD50_RS14355, 14365, and 14375) irrespectively of their own similarity to MtoA or MtrD proteins. Comparatively deep branching of Z-1002 multihemes from those of AHT1^T^ strain was observed for OMD50_RS14375 and OMD50_RS09855 MtrD-like proteins.

#### Dissimilatory reduction of sulfur and thiosulfate

**Figure 5:**
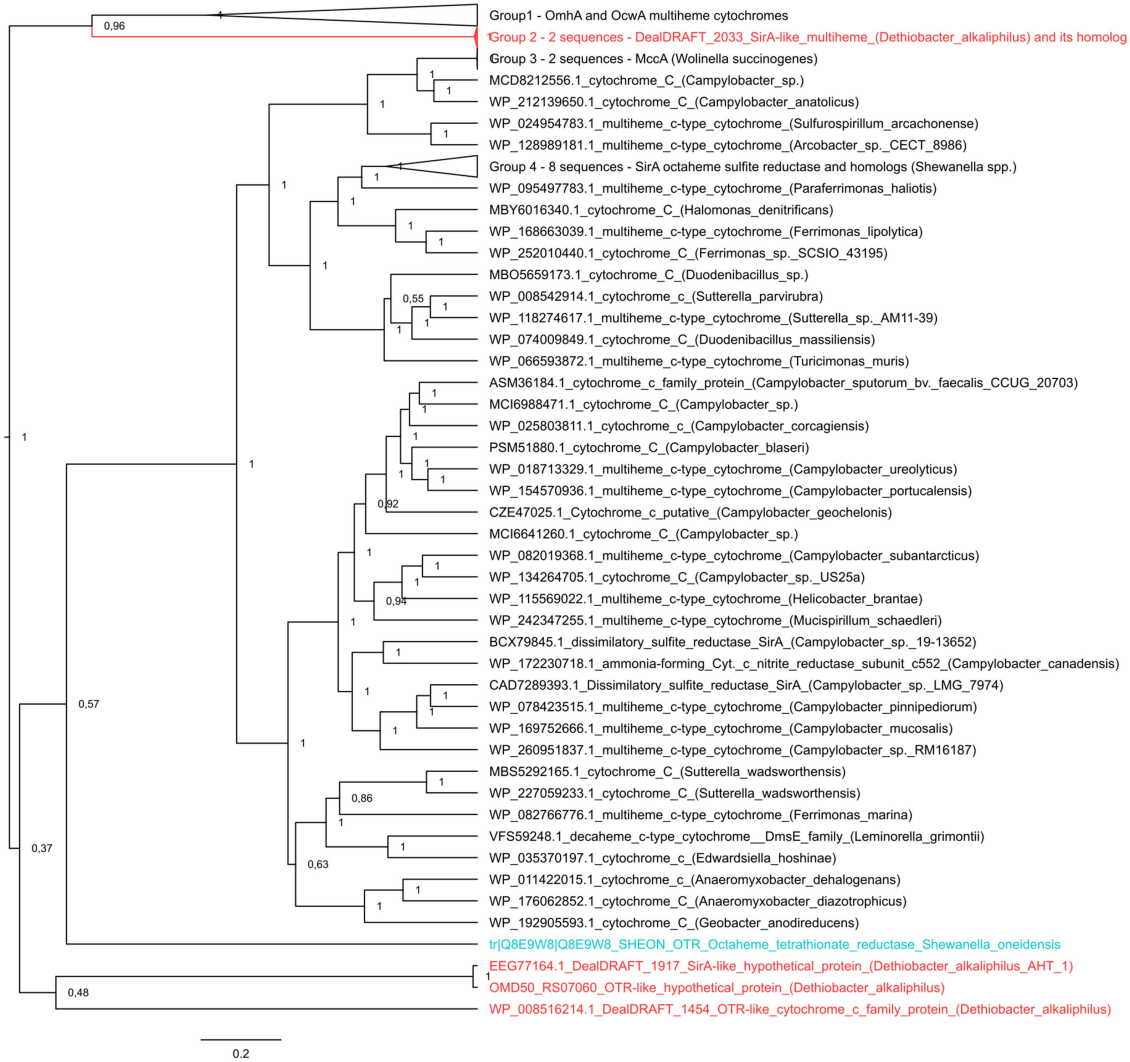
The consensus trees constructed after Bayesian inference of phylogeny from the MAFFT alignment of SirA and Otr cytochromes of *S. oneidensis*, their homologs from *D. alkaliphilus* strains, and best blast hits from public databases. Refer to “Materials and Methods” section for the detailed description of protein selection for the analysis. Homologs of SirA and Otr proteins from *D. alkaliphilus* genomes are summarized in Supplementary Table S2. The unrooted 50% majority rule consensus phylogram is displayed as a rectangular tree, for which posterior probability values are shown. Mean branch lengths are characterized by scale bars indicating the evolutionary distance between the proteins (changes per amino acid position). The branches are annotated with labels indicating the protein sequence accession number, the protein name as retrieved from the database and the source organism. Labels of the proteins are colored *red* for the proteins retrieved from *D. alkaliphilus* strains, *cyan* for the manually added Otr octaheme tetrathionate reductase from *S. oneidensis* and *black* for the other proteins.

It was previously noted that the sulfur- and thiosulfate reducing type strain of *D. alkaliphilus* does not possess any canonical determinants of sulfate, sulfur, or thiosulfate respiration—neither DsrAB complexes, nor molybdopterin oxidoreductases. Instead, only *c*-type multiheme oxidoreductases have been identified in its genome (Melton et al., 2017; Sorokin and Merkel, 2019). Our analysis supported previous assumptions and revealed in both strains the genes of *c*-type cytochromes that are homologous to the Otr class of octaheme tetrathionate reductases (Simon et al., 2011) or SirA/MccA-like sulfite reductases (Kern et al., 2011; Shirodkar et al., 2011) (20 and 22–23% identity, respectively, Supplementary Table S1). In addition, we have identified the clusters encoding [NiFe] hydrogenases of the Group 3b together with beta and gamma sulfur reductase subunits of sulfhydrogenase complexes in both strains. Such enzyme complexes are proposed to harbor the activity, reducing elemental sulfur with molecular hydrogen (Ma et al., 1993). However, strain Z-1002, incapable of sulfur reduction, possesses a sulfhydrogenase cluster too, that raises doubts about the involvement of sulfhydrogenases in sulfur respiration of *D. alkaliphilus*. The difference between the two strains was observed in putative octaheme determinants of sulfur reduction. Each of the strains possesses two cytochromes. These are DealDRAFT_1917 similar to OMD50_RS07060, encoded within identical gene clusters 5-T and 4, respectively, and DealDRAFT_2033 homologous to OMD50_RS05590 as well as to the SirA sulfite reductase of *S. oneidensis.* The clusters 5-T and 4 also encode rhodanese (Cipollone et al., 2007) domain-containing proteins (Figure 4 and Supplementary Table S1). Notably, the protein DealDRAFT_1917 was previously misidentified as a thiosulfate sulfurtransferase (Melton et al., 2017). The sulfur reducing type strain AHT1^T^ possesses an additional homolog of Otr, DealDRAFT-1,454, which is encoded in the cluster 3-Fe-T (Figure 4) and has no homologs among the proteins of strain Z-1002. All these *D. alkaliphilus* multihemes also share homology with previously described EET-related cytochromes (Supplementary Table S1). This fact correlates with recently revealed structural similarity between SirA/MccA octaheme sulfite reductases and OcwA/OmhA terminal Fe(III) reductases (Soares et al., 2022). Phylogenetic reconstruction of all the SirA and Otr homologs from *D. alkaliphilus*, together with its OmhA and OcwA homologs taken as an outgroup, revealed a deep branching of the octahemes from a common ancestor of both Otr- and SirA-like proteins. Interestingly, the homologous proteins DealDRAFT_2033 and OMD50_RS05590 clustered together with the OcwA and OmhA Fe(III) reductases and branched off all the other analyzed cytochromes (Figure 5). The evolutionary history of the octahemes from *D. alkaliphilus* is likely to include their early branching off the common ancestor of Otr- and SirA-like proteins with rather rapid further separation of the DealDRAFT_1,454 protein and the ancestor of highly similar DealDRAFT_1917 and OMD50_RS07060 cytochromes.

### Proteomic profiling of *Dethiobacter alkaliphilus* cells

To assess the relevance of the identified multihemes for EET and to detect any accessory proteins involved in electron transfer to Fe(III) or sulfur compounds, as well as from Fe(II) minerals, the results of a shotgun proteomic analysis of cells, harvested at late logarithmic growth phase, were compared across two different cultivation conditions for each strain. The strain AHT1^T^ was cultured with ferrihydrite or thiosulfate, and the strain Z-1002 was cultured with ferrihydrite or the mixture of Fe(II) minerals (siderite, green rust, and iron oxides). In each case, the triplicate samples were taken from three different cultures (in three biological replicas).

**Figure 6:**
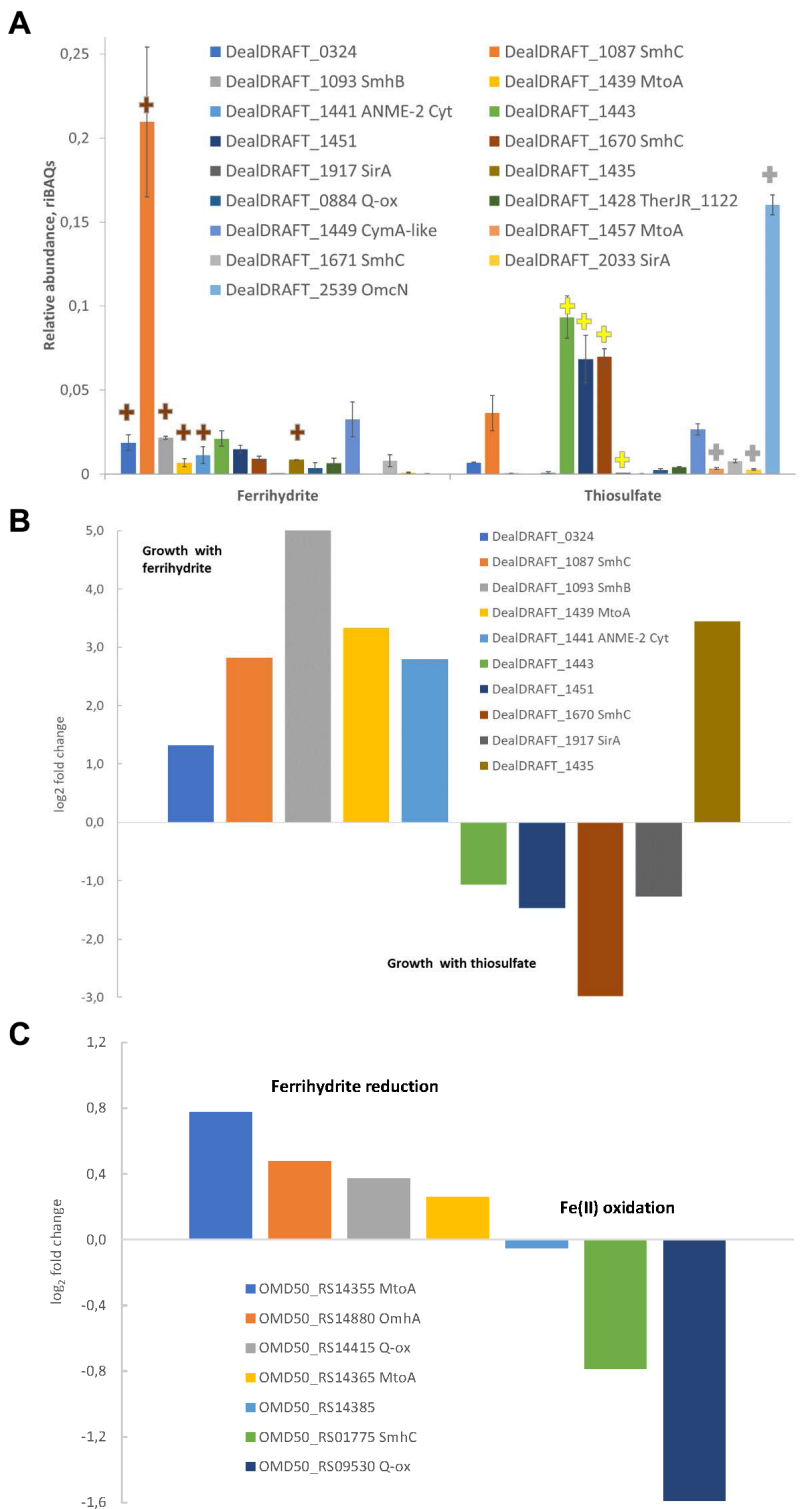
Differential expression profiles of multiheme cytochromes of *D. alkaliphilus* strains. For the strain AHT1^T^, **(A,B)** ferrihydrite- and thiosulfate-grown cells are compared. For the strain Z-1002, Fe(III) reducing and Fe(II) oxidizing cells are compared. **(A)** Molar proportions (riBAQs) of individual multiheme cytochrome proteins of AHT1^T^ strain cultures, **(B)** statistically supported upregulation of individual multihemes in ferrihydrite-grown vs. thiosulfate-grown cells of the strain AHT1^T^, **(C)** statistically supported upregulation of individual multihemes in ferrihydrite-reducing vs. Fe(II)-oxidizing cells of the strain Z-1002. **(A)** Presents the data for all the EET-related cytochromes of the strain AHT1^T^, which were identified in protein profiles. *Brown plus signs* on **(A)** mark the cytochromes which upregulation in AHT1^T^ ferrihydrite-grown cells was statistically supported **(B)**, *yellow plus signs* mark reproducibly upregulated proteins in thiosulfate-grown cells **(B)**, *gray plus signs* indicate the proteins which differential expression was not statistically supported. On panel **(B)**, upregulation in thiosulfate grown cells is visualized by negative values of log_2_(fold changes). On panel **(C)**, upregulation in Fe(II)-oxidizing cells of Z-1002 strain is visualized by negative values of log_2_(fold changes). The cytochromes on all the panels are marked with locus tags and their close homologs among reported EET-related proteins (refer to Supplementary Table S1 for details). All the proteomic data were obtained for three biological replicates, mean values and mean deviation are presented on *A* and in the Supplementary Table S3, S4.

In the case of the AHT1^T^ strain, tandem MS revealed 1910 different proteins identified with a least of two unique peptides. The number of proteins identified for each sample is provided in Supplementary Table S3. Profiling of individual multiheme proteins revealed 17 EET-related multihemes that were differentially expressed under different growth conditions. A clear difference in multiheme cytochrome profiles was observed between ferrihydrite- and thiosulfate-grown cells. However, several proteins, such as DealDRAFT_1449 homologous to a CymA-like quinol oxidase (Supplementary Table S1), were expressed at the same level at both culture conditions (Figure 6A). In contrast, the protein DealDRAFT_1,457 appeared to be the only one exclusively expressed in thiosulfate reducing cells (Figure 6A). In ferrihydrite-grown cells of strain AHT1^T^, the highest relative abundance was observed for DealDRAFT-1,087 which is homologous to the SmhC (23% identity) Fe(III)-specific secreted multiheme of *C. ferrireducens* (Supplementary Table S1). In thiosulfate-grown AHT1^T^ cells, the highest expression level was detected for the multiheme DealDRAFT_2,539 (Figure 6A) but in this case, the difference in the expression level was not statistically supported by the imputation procedure due to the absence of DealDRAFT_2,539 protein in one of the three preparations of the thiosulfate-grown biomass. Statistically relevant upregulation was observed for the proteins DealDRAFT_0324, 1087, 1093, 1435, 1439, and 1441 under ferrihydrite reduction and for the proteins DealDRAFT_1443, 1451, 1670, and 1917 (Figure 6B) under thiosulfate reduction. In addition, the cytochrome DealDRAFT_2033 was overexpressed in thiosulfate-grown cells (Figure 6A) but the absence of this protein in two of three preparations of ferrihydrite-grown biomass did not statistically support the differences in its expression level. The majority of the proteins with positive response to ferrihydrite provided as an electron acceptor are encoded in the clusters 1-Fe-T, 2-Fe-T, 3_Fe-T (Figure 4) and share homology with putative terminal Fe(III) reductases, secreted multihemes, and MtoA putative Fe(II) oxidase which is phylogenetically related to MtrA/MtrD Fe(III) oxidases (Shi et al., 2012). Interestingly, four of the six multihemes upregulated in thiosulfate-grown cells (DealDRAFT_1443, 1451, 1670, and 2539) were also homologous to various cytochromes related to Fe(III) reduction (Supplementary Table S1). Only a single protein homologous to SirA sulfite reductase (22% identity), DealDRAFT_1917, was reliably upregulated during *D. alkaliphilus* growth on thiosulfate.

In the strain Z-1002, tandem MS identified 1,390 different proteins identified with at least two unique peptides. The number of proteins identified per each sample is provided in Supplementary Table S4. Profiling of individual multiheme proteins uncovered nine EET-related multihemes that were differentially expressed under ferrihydrite reduction or Fe(II) oxidation. The cytochromes OMD50_RS14355, 14365, 14415, and 14880, homologous to MtoA Fe(II) oxidases, CymA-like quinol oxidase and OmhA terminal Fe(III) reductase, respectively (Supplementary Table S1), had almost similar expression levels under ferrihydrite reduction or Fe(II) oxidation (Figure 6C). The highest expression levels under both Fe(III)-reducing and Fe(II)-oxidizing conditions were observed for the OMD50_RS14385 and OMD50_RS14880 proteins (Supplementary Table S4) which are homologous to cell surface-associated cytochromes of *C. ferrireducens* (30 and 26% identity, respectively, Supplementary Table S1). These proteins are likely to comprise the core part of the EET pathway driving the redox transformation of Fe minerals in *D. alkaliphilus*. Interestingly, the cytochromes OMD50_RS02905 and 7,060, homologous to OmhA Fe(III) reductase and SirA sulfite reductase (25 and 23% identity, respectively) were exclusively expressed under Fe(II) oxidation, whereas a putative quinol oxidase OMD50_RS09530 showed the strongest upregulation under the same growth conditions (Figure 6C).

### Enrichment of *Dethiobacter* representatives from subsurface mineral waters of YMWD

**Figure 7:**
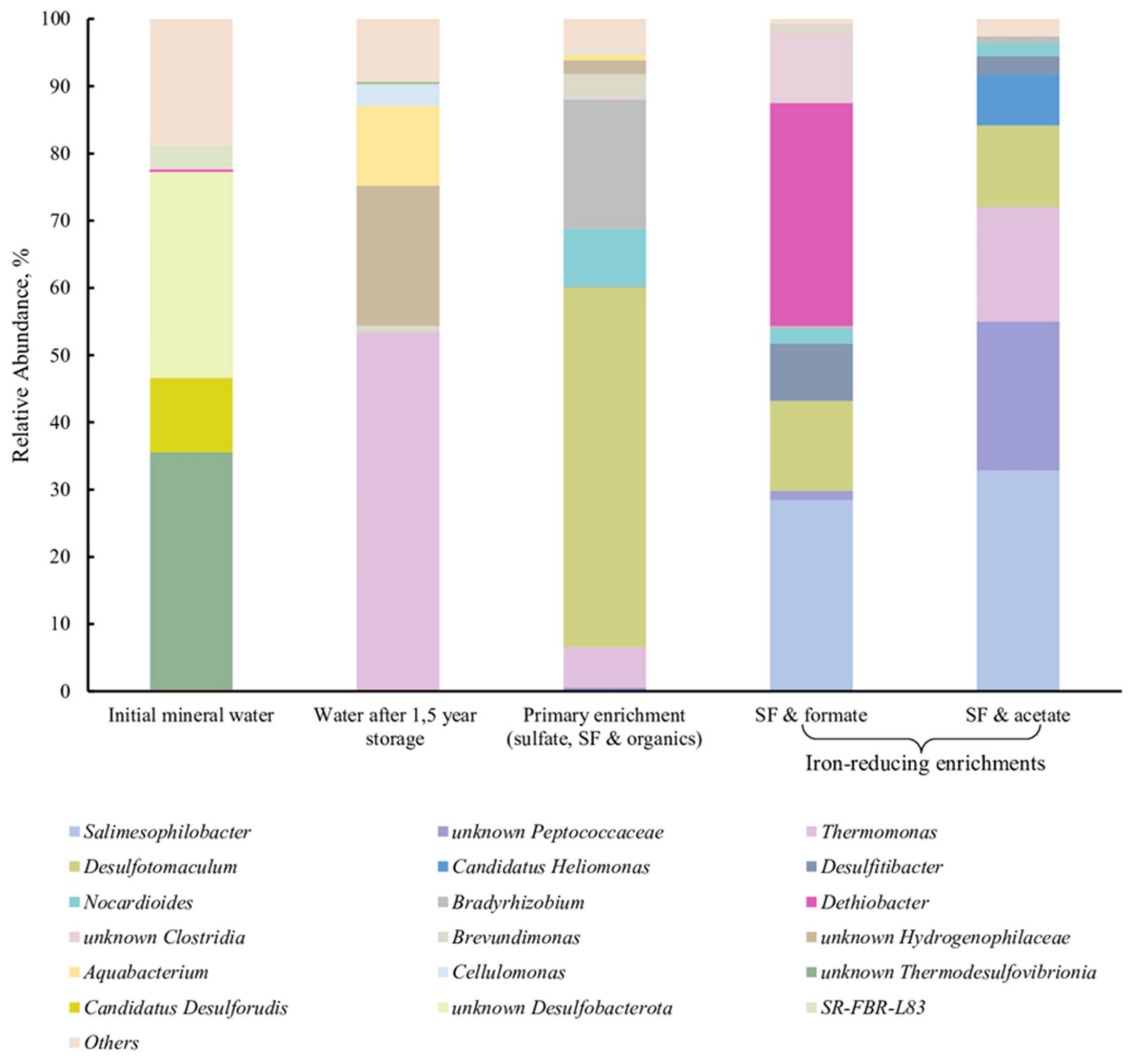
16S rRNA gene-based profiling of environmental samples and enrichment cultures from subsurface mineral water of the Lower Cretaceous aquifer of YMWD.

We have detected a phylotype belonging to the genus *Dethiobacter* in a subsurface mineral water sample collected from the Lower Cretaceous aquifer of YMWD through well 9. The phylotype comprised less than 0.1% of the microbial diversity of this sample (Figure 7, 1st column). The sample was then stored at +4°C for 1.5 years and then used for the enrichment of prokaryotes inhabiting the deep subsurface aquifer using a mixture of electron donors and acceptors available in this environment. This primary enrichment containing both sulfate and ferrihydrite (as SF) and incubated at +30°C for 3 weeks was dominated by typical sulfate reducing taxa but carried out the reduction of SF to a black precipitate. No *Dethiobacter* representatives were detected in the water sample after its long-term storage (1.5 years) or in the primary enrichment at the end of its 3-weeks incubation (Figure 7, 2nd and 3rd columns). To check the presence of iron reducers in the obtained enrichment, it was transferred to selective media containing formate (10 mM) or acetate (10 mM) with SF (50 mM final Fe(III) content) as the only electron acceptor. After 2 weeks of incubation, the color of the Fe mineral changed from reddish to dark-brown, which is characteristic of ferrihydrite reduction. Fluorescence microscopy of mineral samples taken from these cultures revealed that mineral particles were densely settled with rod-shaped cells of different length. 16S rRNA gene profiling of the Fe(III) reducing enrichments revealed the predominance of *Dethiobacter* phylotypes (96% identity with *D. alkaliphilus*) in the culture with SF and formate (33.2% relative abundance) and their complete absence in the culture with SF and acetate (Figure 7, 4th and 5th columns, respectively).

### Environmental distribution of *Dethiobacter*-related phylotypes

The survey of the environmental distribution of 16S rRNA gene sequences of the genus *Dethiobacter* available in public databases shows that about 25% of the sequences are retrieved from sediments of soda lakes and alkaline soils, 35% of the sequences are retrieved from Fe-enriched serpentinites, about 30%—from anaerobic bioreactors and digesters (Supplementary Figure S5). The remaining small fraction of sequences was found in microbial mats, ground and urban waters. Thus, *Dethiobacter* is the most abundant in sulfur- or iron-enriched alkaline natural habitats. However, the representatives of the genus have also been detected in anthropogenic organics-rich neutrophilic environments. Unfortunately, the lack of publicly available data on the physico-chemical conditions sustained in these anthropogenic environments, and on the sources of wastewaters treated there do not allow us to discuss the phylogenetic group of *Dethiobacteraceae* detected in bioreactors in the context of the evolution of their metabolic capacities.

## Discussion

Currently, *Dethiobacter alkaliphilus*, the only cultured representative of the class “*Dethiobacteria*,” harbors two strains with contrasting adaptation to the conditions favoring iron or sulfur cycling. The type strain is a sulfidogen capable of elemental sulfur reduction (Sorokin et al., 2008), as well as of chemolithoautotrophic sulfur disproportionation (Poser et al., 2013, 2016), while strain Z-1002 appears to be incapable of sulfur reduction at all. However, both strains are capable of thiosulfate reduction (Table 2). Comparative genome analysis revealed the background of these physiological differences.

**Table 2:** Phenotypic and genotypic differences between the two strains of *Dethiobacter alkaliphilus*.

Characteristics	Strain AHT1^T^	Strain Z-1002
pH range (optimum)	8.5–10.3 (9.5)^1^	7.8–10.1 (9.2)
Na^+^ range (optimum), M	0.2–1.8 (0.4)^1^	0.35–2.5 (1.0)
S^0^ disproportionation	+^2^	−
S_2_O_3_^2−^ disproportionation	+^2^	−
S^0^ reduction	+^1^	−
S_2_O_3_^2−^ reduction	+^1^	+
Fe(III) reduction	+	+^3^
Fe(II) oxidation	−	+
Genome size (bp)	3,116,746	3,235,311
Protein-coding genes	3,097	3,225
Multiheme cytochrome genes	31	27
Putative gene clusters involved in Fe cycling^4^	4	3
Homologs of MtoA Fe(II) oxidases^4^	3	4
Homologs of SirA- and Otr-like octaheme cytochromes^5^	3	2

^1^Sorokin et al. (2008), ^2^Poser et al. (2013), Poser et al. (2016), ^3^Zavarzina et al. (2018), ^4^refer to Supplementary Table S1 for details; ^5^refer to Figure 5 for details.

### Non-canonical multiheme determinants of thiosulfate reduction in *Dethiobacter alkaliphilus*

Both *D. alkaliphilus* strains appeared to lack canonical genomic determinants of sulfur, sulfate or thiosulfate reduction, such as DsrAB or molybdopterin-containing complexes. Instead, each of the strains possessed two octaheme *c*-type cytochromes homologous to the SirA sulfite reductase or Otr tetrathionate reductase of *S. oneidensis* (Supplementary Table S1). The sulfur-reducing type strain possesses an additional weak homolog of Otr proteins, DealDRAFT_1454 (20% identity). Phylogenetic reconstruction of these putative determinants of sulfur compounds reduction revealed deep branching of three of them from the common ancestor of SirA and Otr proteins, and association of two *D. alkaliphilus* proteins with OcwA/OmhA Fe(III) reductases (Figure 5), which are structurally similar to, but phylogenetically distant from octaheme sulfite or tetrathionate reductases (Soares et al., 2022).

Multiheme cytochrome profiling revealed upregulation of the protein DealDRAFT_1917, homologous to SirA, in the cells of the strain AHT1^T^ grown with thiosulfate (Figure 6). Interestingly, the protein DealDRAFT_1457 homologous to MtoA Fe(II) oxidase was only detected in thiosulfate-grown cells. Furthermore, the most pronounced upregulation in thiosulfate-reducing cells was observed for DealDRAFT_1451 and 1670 (Figure 6) homologous to the putative Fe(III) reductases SmhB and SmhC (21 and 32% identity, respectively, Supplementary Table S1). The highest expression level in these cells was detected for the protein DealDRAFT_2539 homologous (24% identity) to OmcN Fe(III)-reducing outer surface cytochrome of *G. sulfurreducens* (Aklujkar et al., 2013). All these facts emphasize that the reduction of sulfur compounds in *D. alkaliphilus* is most likely driven by multiheme cytochromes homologous to Fe-cycling proteins.

### Multiheme determinants of redox transformations of iron in *Dethiobacter alkaliphilus*

We have experimentally demonstrated the ability of the sulfur-reducing strain AHT1^T^ to reduce Fe(III) from SF and the ability of the Fe(III)-reducing strain Z-1002 to oxidize Fe(II) from green rust supplied as a mixture with siderite. The result of Fe(II) oxidation by strain Z-1002 was indicated by a 6.4% increase in the relative intensity of the Mössbauer spectra of Fe^3+^ atoms compared to that of uninoculated controls (Table 1).

Genomic analysis revealed that each of the *D. alkaliphilus* strains contained a large set of genes encoding multiheme *c*-type cytochromes, including those homologous to quinol-oxidases, membrane-associated Fe(III) reductases, such as OcwA or MtrA/D, and soluble electron shuttling cytochromes, previously identified in the model Fe(III)-respiring bacteria of the genera *Shewanella* and *Geobacter* or in Gram-positive thermophilic Fe(III) reducers (Figure 4 and Supplementary Table S1). Taken together, these cytochromes could be combined in an electron transfer chain linking the membrane-bound respiratory complexes with extracellular electron acceptors, such as Fe(III)-containing minerals, or electron donors, such as Fe(II)-bearing mineral mixtures. Proteomic profiling of strain AHT1^T^ for multihemes revealed strong upregulation of six different cytochromes, including the cytochrome DealDRAFT_1439 (Figure 6), encoded in three gene clusters of putative Fe(III) reductases (clusters 1-Fe-T, 2-Fe-T, 3-Fe-T, Figure 4). These cytochromes contain the homologs of all the necessary components of an extracellular electron transfer chain to Fe(III) oxides. Interestingly, the Fe(II)-oxidizing strain Z-1002 possesses four different homologs of the protein DealDRAFT_1,439 which are encoded in the 2-Fe and 3-Fe clusters. Of these, the proteins OMD50_RS14355 and OMD50_RS14365 share similarity with MtoA-type Fe(II)-oxidases (Shi et al., 2012). Phylogeny reconstruction of these and other MtoA homologs of *D. alkaliphilus* (DealDRAFT_1454, 1457, OMD50_RS14520) revealed clear separation of these proteins from DmsE-family decahemes of other organisms that perform Fe(III) reduction or Fe(II) oxidation (Supplementary Figure S4). Branching most deeply from the common ancestor of all the *D. alkaliphilus* decahemes is the phylogenetic cluster of MtoA-like cytochromes DealDRAFT_1439, OMD50_RS14355, and OMD50_RS14365 (Supplementary Figure S4), as well as the protein OMD50_RS14375 with closer homology (24% identity) to the MtrD Fe(III)-reducing cytochrome of *S. oneidensis* (Shi et al., 2012). We propose that three of the four proteins in this phylogenetic cluster might appear in strain Z-1002 by several duplication events with further minor changes occurred under the evolutionary pressure of the conditions favoring Fe-cycling, such as increased abundance of mixed Fe(III)/Fe(II)-containing minerals. Such an assumption correlates with the similarity of expression levels of two MtoA-like cytochromes in strain Z-1002 cells grown under ferrihydrite reducing or Fe(II) oxidizing conditions (Supplementary Table S4 and Figure 6C). In general, multi-omics analysis revealed that the biochemical machinery of Fe redox cycling in *D. alkaliphilus* strains is more complex and flexible than that of sulfur respiration.

### Junctions of the pathways for iron and sulfur respiration in *Dethiobacter alkaliphilus*

The most likely quinol oxidizing/quinone reducing proteins of the respiratory chains in both *D. alkaliphilus* strains are the CymA-like homologs DealDRAFT_1449 and OMD50_RS14415 with their stable expression level almost independent of the electron acceptors or donors provided (Figure 6). Importantly, the majority of the cytochromes overexpressed under ferrihydrite- or thiosulfate-reducing conditions in the strain AHT1^T^ are encoded in three largest gene clusters (Figure 4), which also encode auxiliary flavin-containing electron transfer proteins together with NHL- and TPR-repeat-containing proteins that could be involved in protein–protein interactions mediating the assembly of multiprotein complexes (D’Andrea and Regan, 2003), such as MtrCAB Fe(III)-reducing ones (Shi et al., 2012). Interestingly, the putative Fe-cycling multiheme proteins of *D. alkaliphilus* showed greater divergence from each other than that observed among the EET-related proteins of the DmsE family from fairly different bacteria. Another important point is that the homologs of OmhA Fe(III) reductase and SirA sulfite reductase are expressed only under Fe(II) oxidation in strain Z-1002. These facts highlight the intricate weaving of EET pathways, that determine the redox transformations of sulfur and iron compounds in the organism, which could be a manifestation of the active evolution of multiheme proteins within *D. alkaliphilus* species, characteristic of the ongoing adaptation of an evolving population to the changes in its environment (Zheng et al., 2019).

### Proposal of an adaptation strategy of *Dethiobacter alkaliphilus* to geochemical settings of soda lakes

Our analysis of the distribution of *Dethiobacters* in natural environments revealed that the representatives of this genus show strong addiction to two peculiar alkaline, sulfur- or iron-enriched ecotopes, soda lakes and serpentinite-associated sediments, respectively (Supplementary Figure S5). Serpentinizing environments have been stable throughout the Earth’s geological evolution, whereas soda lakes are subjected to continuous biogeochemical development, being surfaceous ecosystems (Stüeken et al., 2015; Furnes and Dilek, 2022). Their most pronounced restructuring occurred after the Great Oxidation Event followed by the accumulation of sulfates in the sediments and waters of the lakes (Montinaro and Strauss, 2016). Since then, the biogeochemical cycling of sulfur has become one of the major factors determining the physicochemical conditions in soda lakes and influencing the evolution of their inhabitants.

The phylogenetic reconstruction of the determinants of key catabolic processes, sustaining the growth of two *D. alkaliphilus* strains (Figure 5 and Supplementary Figure S4), offers insights into the evolutionary traits which led to the occupation of two different ecological niches by this bacterial species. The presence of multiheme cytochromes, sharing their phylogenetic root with OmhA/OcwA Fe(III)-reductases, as the only possible determinants of the reduction of sulfur compounds in *D. alkaliphilus* allows us to propose that soda lakes are secondary habitats for these organisms comparing to Fe-rich subsurface environments associated with serpentinites. This hypothesis correlates with the geological history and current geochemical characteristics of Magadi soda lake from which the Fe-reducing strain Z-1002 was isolated. This lake is located in a geologically young East African Rift Valley characterized by high pH, silica and carbonate concentration. The ecotope was formed *ca.* 1 million years ago due to faulting of the Rift Valley composed of alkali Pleistocene basalt, trachyte lava flows and phonolite (Jones et al., 1977; Eugster, 1980; Schagerl and Renaut, 2016). Thus, the bedrock of the modern lake appeared to be enriched with iron minerals. In this case, the Fe-reducing *D. alkaliphilus* could be an alien species for lacustrine sulfur-rich sediments, which was introduced there from the underlying Fe-rich volcanic rocks. The strain was managed to modify its multiheme cytochrome machinery under the evolutionary pressure of renewed geochemical settings with predominant sulfur cycle. A large number of different multiheme cytochrome genes and complete absence of typical genes determining the redox transformations of sulfur compounds could favor the adaptation of *Dethiobacter* to an ecotope where Fe-rich rocks are combined with sulfur-enriched sediments and waters.

Better fitness of the metabolic features of *Dethiobacters* to the life in Fe-rich alkaline environments was highlighted by the enrichment of their phylotypes from the Lower Cretaceous aquifer of the Yessentukskoye subsurface mineral water deposit (YMWD). The crystalline basement of the deposit is represented by Proterozoic-Paleozoic metamorphic and magmatic shales and granites, i.e., the rocks depleted with iron-bearing minerals. The sedimentary cover of the YMWD is represented by iron-depleted limestones, mudstones and siltstones of Meso-Cenozoic age. The Lower Cretaceous aquifer of the YMWD, penetrated by the well 9 which we have sampled, is directly connected with the recharge area and contains carbon-free sulfaceous alkaline waters (Elena et al., 2020; Filimonova et al., 2022). This environment is completely different from those formed by serpentinization processes. Not surprisingly, *Dethiobacter*-related phylotypes comprised a minor part (0.08%) of the microbial community in this aquifer and rather represented the so-called “rare biosphere,” which is considered a phenotypic repository of microbial communities getting advantages upon significant changes of environmental conditions (Jousset et al., 2017). In our experiments, *Dethiobacter* phylotypes appeared to pass undetected through a long-term storage of a mineral water sample and its further cultivation under sulfate-reducing conditions. The phylotypes restored when the enrichment conditions became favorable for alkaliphilic lithotrophic Fe(III) reducers. In this case, *Dethiobacter* representatives became the dominant group of the enrichment culture (Figure 7). Thus, Fe-rich alkaline conditions seem to be optimal geochemical settings for yet uncultured *Dethiobacter* species. This fact together with the unity of origin of the sulfur, thiosulfate and Fe(III) reduction pathways in *D. alkaliphilus*, allows us to propose an adaptation strategy of the organism to the change of its environment from serpentinizing Fe-rich ecotopes to soda lakes. This strategy is based on the changes in the multiheme cytochrome repertoire aimed to get energy from the reduction of electron acceptors with lower redox potential (sulfur compounds vs. Fe(III) minerals). Such an example of intraspecific microevolution within *D. alkaliphilus* shows a possible way of a global adaptive response of prokaryotes to the activation of the sulfur cycle after the appearance of sulfates in the oceanic water and free oxygen in the atmosphere during GOE (Catling and Zahnle, 2020).

### Emended description of *Dethiobacter alkaliphilus*

In addition to the characteristics given for the type strain of the species, AHT1^T^ (Sorokin et al., 2008; Poser et al., 2013, 2016; Sorokin and Merkel, 2019), and strain Z-1002 (Zavarzina et al., 2018), the following characteristics should be added to the formal description: both strains of the type species can grow by iron-reduction in the presence of molecular hydrogen, formate, acetate, lactate, succinate, pyruvate, butyrate, propionate or ethanol as the electron donors. Strain Z-1002 is unable to reduce elemental sulfur or disproportionate thiosulfate and elemental sulfur. Strain Z-1002 is able to reduce thiosulfate or anaerobically oxidize Fe(II)-containing minerals.

## Data availability statement

The datasets presented in this study can be found in online repositories. The names of the repository/repositories and accession number(s) can be found at: https://www.ncbi.nlm.nih.gov/genbank/, JAPDNO000000000.

## Author contributions

DZ, AYM, AK, VP, VR, and NC: experimental work. DZ, AAM, and SG: field sampling. AYM and SG: genome annotation and analysis. AYM and IE: phylogenetic analyses. VR and NC: Mössbauer spectroscopy and analysis. RZ and VP: proteomic studies. RZ: modification of routine protocols for the samples with low protein content, LC-MS/MS analysis, data processing and statistical analysis. DZ, SG, AYM, and AAM: writing the manuscript. DZ: convene the research. SG and AAM: acquire funding. All authors contributed to the article and approved the submitted version.

## Funding

This research was partially funded by the Russian Science Foundation (grant no. 21-14-00333) (DZ, SG, and VP, physiological studies, enrichments, genome analysis), and by the Ministry of Science and Higher Education of the Russian Federation (AK, IE, and AYM, genome sequencing, phylogenetic analysis).

## Conflict of interest

The authors declare that the research was conducted in the absence of any commercial or financial relationships that could be construed as a potential conflict of interest.

## Publisher’s note

All claims expressed in this article are solely those of the authors and do not necessarily represent those of their affiliated organizations, or those of the publisher, the editors and the reviewers. Any product that may be evaluated in this article, or claim that may be made by its manufacturer, is not guaranteed or endorsed by the publisher.

## Footnotes

^1^https://www.ebi.ac.uk/Tools/hmmer/search/hmmscan
