# Supplementary material for: Iron or sulfur respiration—an adaptive choice determining the fitness of a natronophilic bacterium Dethiobacter alkaliphilus in geochemically contrasting environments
Source: Front Microbiol. 2023 Jul 14;14:1108245. doi: 10.3389/fmicb.2023.1108245 (PMC10376724; doi:10.3389/fmicb.2023.1108245)
Supplement: Supplementary file 1 [file Data_Sheet_1.DOCX]

**Iron or sulfur respiration – an adaptive choice determining the fitness of a natronophilic bacterium *Dethiobacter alkaliphilus* in geochemically contrasting environments**

Daria G. Zavarzina, Alexander Yu. Merkel, Alexandra A. Klyukina, Ivan M. Elizarov, Valeria A. Pikhtereva, Vyacheslav S. Rusakov, Nataliya I. Chistyakova, Rustam H. Ziganshin,

Alexey A. Maslov, and Sergey N. Gavrilov

**SUPPLEMENTARY MATERIALS**


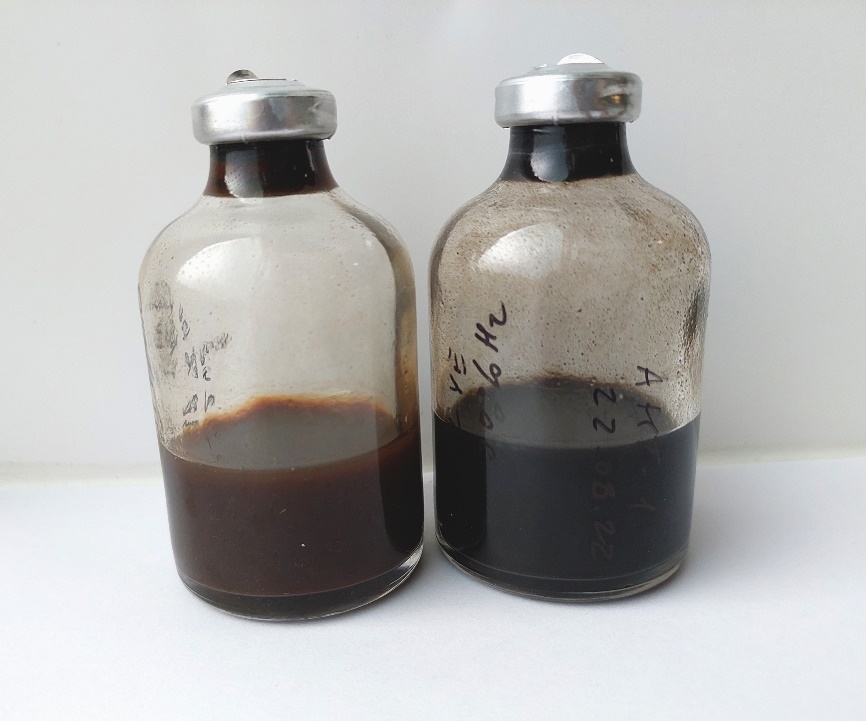


A

B

**Supplementary Figure S1.**

**Color change of the mineral phase during the growth of strain AHT1^T^ with synthesized ferrihydrite and molecular hydrogen after three consequent transfers on the same medium.**

(*A*) – sterile control; (*B*) – grown culture.

**Supplementary Figure S2.**

**Kinetics of the growth of strain Z-1002 during Fe(II) oxidation from a siderite-based mineral mixture.**

The culture was sampled for Mössbauer studies (see **Supplementary Figure S3** below) at a starting point and the stationary growth phase (34^th^ hour of incubation). Cells were directly counted using fluorescent microscopy (refer to the Materials and Methods section for details). Five biological replicas were used for each time point.


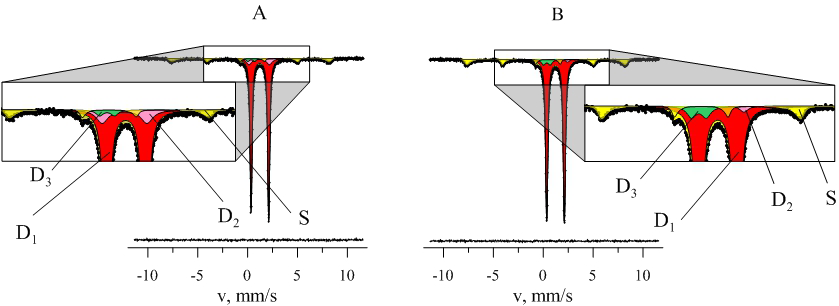


**Supplementary Figure S3.**

**Mössbauer spectra measured at room temperature for the siderite-based mineral mixture incubated *(A)* in a sterile control medium, and *(B)* with the growing culture of strain Z-1002.**

The parameters of quadrupole doublet D_1_ (colored in red) and D_2_ (colored in pink) correspond to ferrous iron. The third subspectrum D_3_ (colored in green) corresponds to ferric ions. Hyperfine parameters of D_1_ are typical for Fe^2+^ atoms in the structure of siderite. The doublets D_2_ and D_3_ most likely correspond to Fe^2+^ and Fe^3+^ atoms in the green rust structure. Note the subspectra corresponding to Fe^3+^ atoms in magnetically ordered phases (iron oxides) in the spectra retrieved from both the control sample and the grown culture.

**
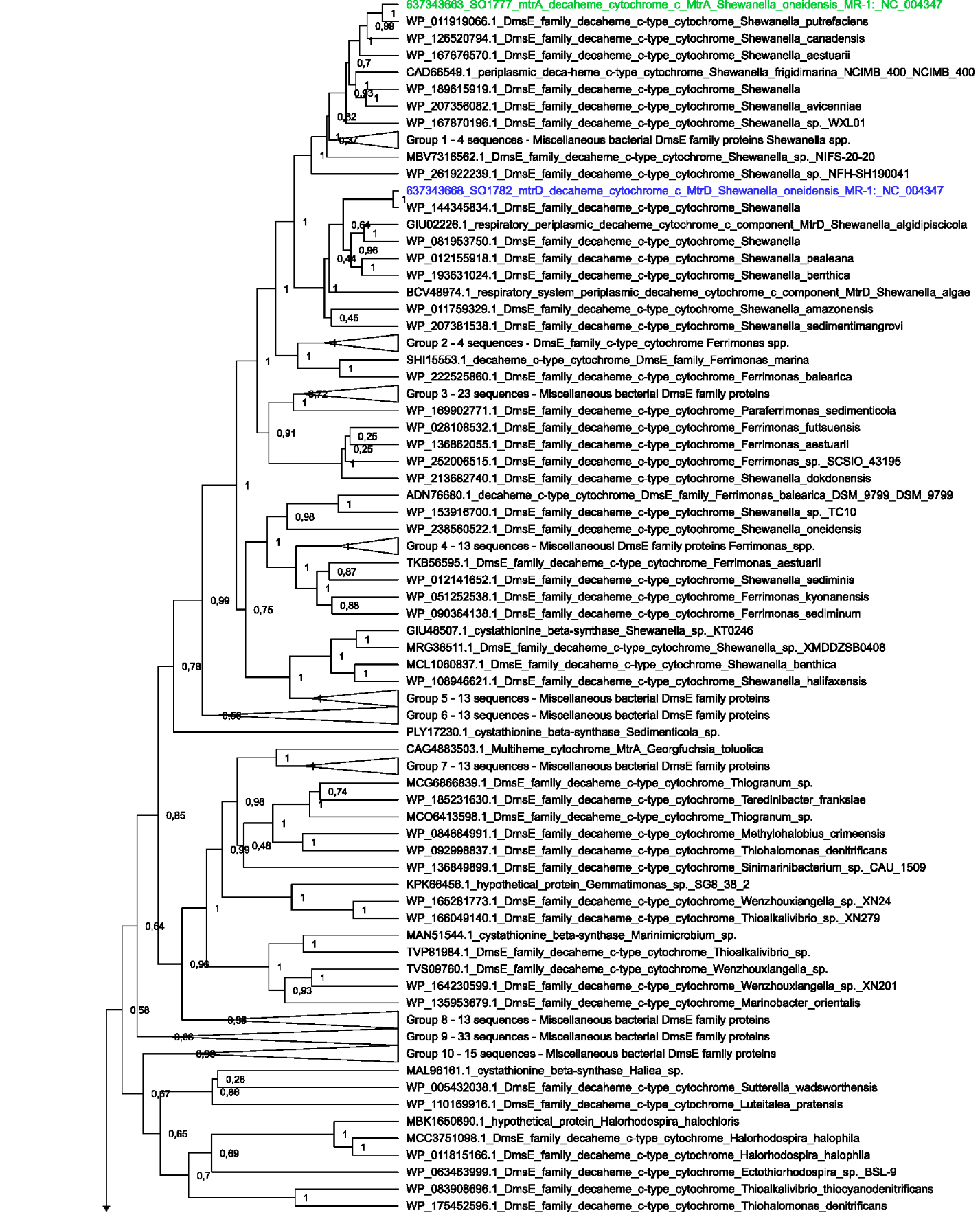
**

**Supplementary Figure S4** | Continued

**
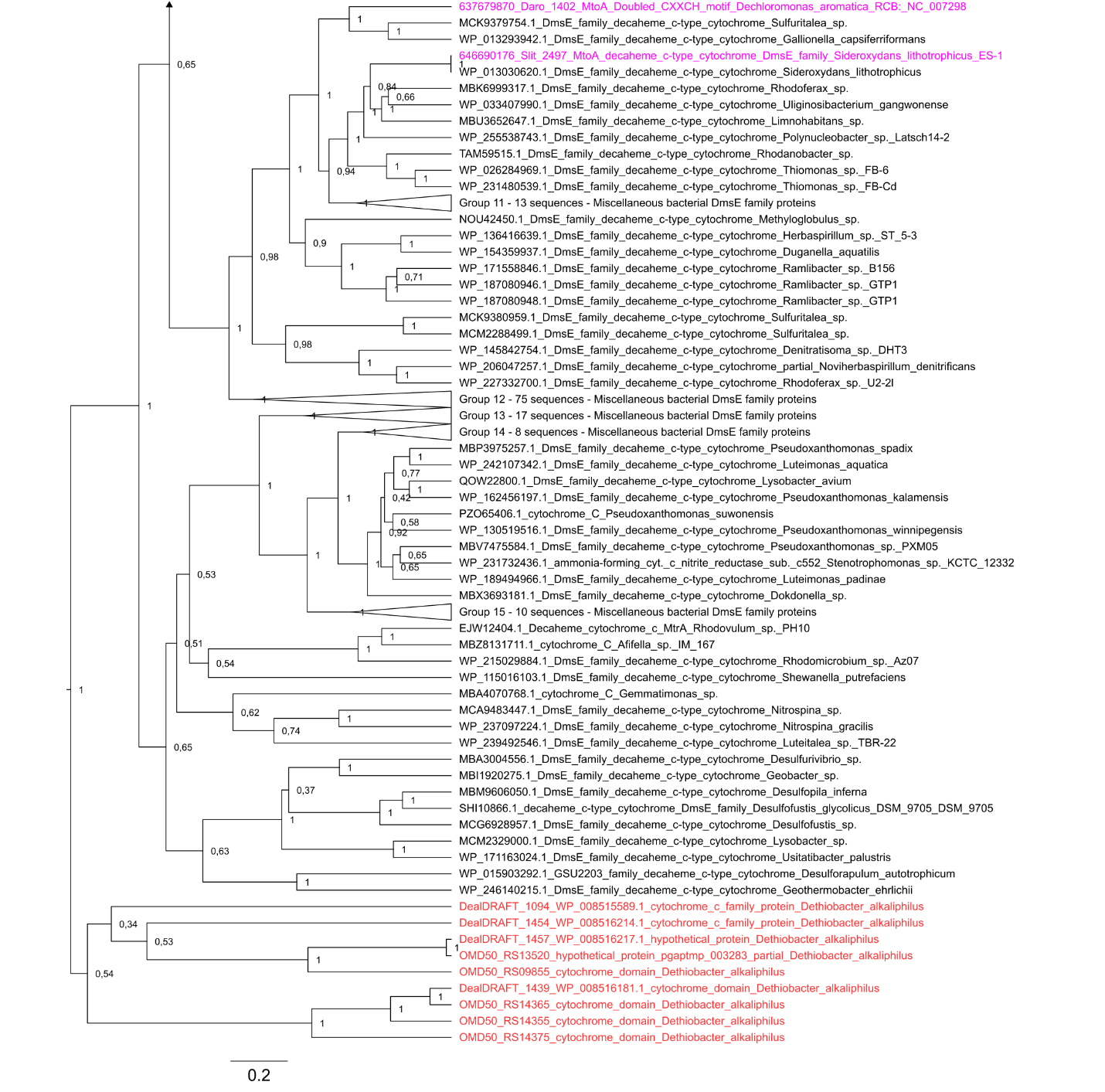
**

**Supplementary Figure S4** | Continued

**Supplementary Figure S4**.

**Consensus tree constructed after Bayesian inference of phylogeny from the MAFFT alignment of the MtoA cytochrome of *G. capsiferriformans*,** **its close homologs from *D. alkaliphilus* strains, and best blast hits from public databases.**

Refer to “Materials and Methods” section for the detailed description of proteins selection for the analysis. Homologs of MtoA proteins from *D. alkaliphilus* genomes are summarized in Supplementary Table S2. The unrooted 50% majority rule consensus phylogram is displayed as a rectangular tree, for which posterior probability values are shown. Mean branch lengths are characterized by scale bars indicating the evolutionary distance between the proteins (changes per amino acid position). The branches are annotated with labels indicating the protein sequence accession number, the protein name as retrieved from the database and the source organism. Labels of the proteins are colored *red* for the proteins retrieved from *D. alkaliphilus* strains, *green and blue* for the manually added functionally characterized proteins from *S. oneidensis* MR-1 (see “Materials and Methods” section for detail), *purple* for the proteins acquired via BLAST and annotated as MtoA cytochromes and *black* for other proteins.


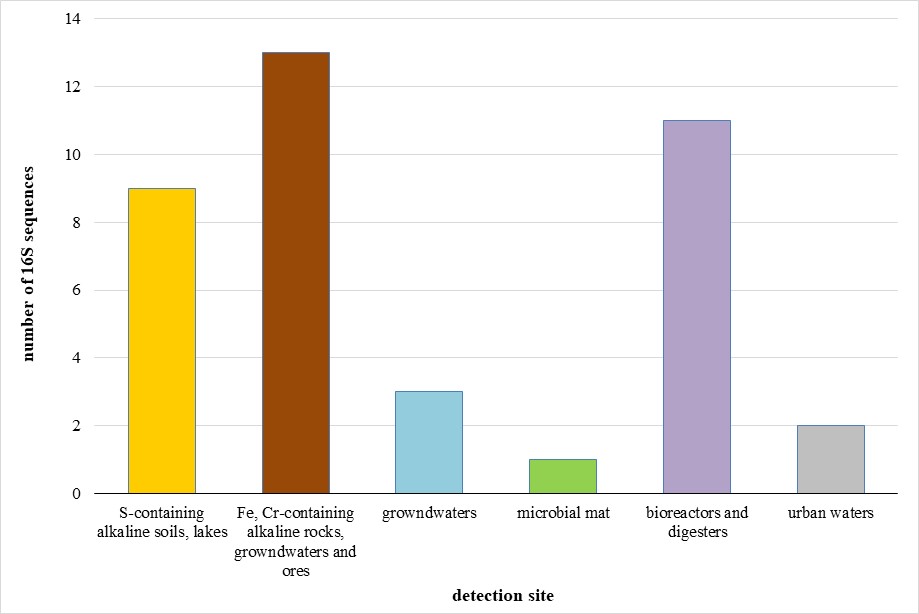


**Supplementary Figure S5.**

**Distribution of *Dethiobacter*-related 16S rRNA gene sequences by their detection sites.**

**Supplementary Tables**

Table S1. Best blast hits of *D. alkaliphilus*' cytochromes among previously reported components of EET pathways in prokaryotes.

Provided as a separate MS Excel file.

Table S2. Genome statistics of the two strains of *D. alkaliphilus.*

| **Assembly parameters** | **Strain AHT1^T^** | **Strain Z-1002** |
| --- | --- | --- |
| Genome Size (bp) | 3116746 | 3235311 |
| Contig Count | 34 | 39 |
| N50 (bp) | 191499 | 232795 |
| CheckM ^1^ Completeness (%) | 95.76 | 96.61 |
| CheckM Contamination (%) | 1.13 | 1.98 |
| Genes (total) | 3196 | 3283 |
| Genes (protein coding) | 3097 | 3225 |
| Genes (RNA) | 64 | 50 |
| rRNAs | 5, 4, 7 (5S, 16S, 23S) | 1, 2 (16S, 23S) |
| tRNAs | 44 | 42 |
| GC (%) | 48.46 | 48.29 |

^1^ Parks DH, Imelfort M, Skennerton CT, Hugenholtz P, Tyson GW. CheckM: assessing the quality of microbial genomes recovered from isolates, single cells, and metagenomes. *Genome Res.* 2015 Jul;25(7):1043-55.

Table S3. Shotgun proteomic profiles of *D. alkaliphilus* strain AHT1^T^ grown with ferrihydrite or thiosulfate as the electron acceptors as determined by LC-MS/MS.

Provided as a separate MS Excel file.

Table S4. Shotgun proteomic profiles of *D. alkaliphilus* strain Z-1002 grown under ferrihydrite reducing or Fe(II) oxidizing conditions as determined by LC-MS/MS.

Provided as a separate MS Excel file.
